# Respiratory syncytial virus in the Western Pacific Region: a systematic review and meta-analysis

**DOI:** 10.7189/jogh.09.020431

**Published:** 2019-12

**Authors:** Krisna N A Pangesti, Moataz Abd El Ghany, Alison M Kesson, Grant A Hill-Cawthorne

**Affiliations:** 1 School of Public Health, Faculty of Medicine and Health, The University of Sydney, Australia; 2 The Westmead Institute for Medical Research, The University of Sydney, Sydney, Australia; 3 Center for Research and Development of Biomedical and Basic Health Technology, NIHRD, Jakarta, Indonesia; 4 Marie Bashir Institute of Infectious Diseases and Biosecurity, The University of Sydney, Sydney, Australia; 5 The Westmead Clinical School, Faculty of Medicine and Health, The University of Sydney, Sydney ,Australia; 6 Discipline of Child and Adolescent Health, The University of Sydney, Sydney, Australia; 7 The Children Hospital at Westmead, Department of Infectious Diseases and Microbiology, Sydney, Australia

## Abstract

**Background:**

Respiratory syncytial virus (RSV) is the leading cause of viral pneumonia and bronchiolitis, especially in younger children. The burden of RSV infection in adults, particularly in the older age group, is increasingly recognised. However, RSV disease burden and molecular epidemiology in the World Health Organization (WHO) Western Pacific Region (WPR) has not been reviewed systematically. The aim of this systematic review is to investigate the epidemiological aspects of RSV (incidence, prevalence, seasonality and hospitalisation status) and the associated molecular data in the WPRO countries.

**Methods:**

A systematic search was conducted in international literature databases (MEDLINE, EMBASE, Scopus and Web of Science) to identify RSV-related publications from January 2000 to October 2017 in the WPR countries.

**Results:**

A total of 196 studies from 15 WPR countries were included. The positivity rate for RSV among respiratory tract infection patients was 16.73% (95% confidence interval (CI) = 15.12%-18.4%). The RSV-positive cases were mostly found in hospitalised compared with outpatients (18.28% vs 11.54%, *P* < 0.001), and children compared with adults (20.72% vs 1.87%, *P* < 0.001). The seasonality of RSV in the WPR countries follows the latitude, with the peak of RSV season occurring in the winter in temperate countries, and during the rainy season in tropical countries. The molecular epidemiology pattern of RSV in WPR countries was similar to the global pattern, with NA1 (RSV A) and BA (RSV B) being the predominant genotypes.

**Conclusions:**

The available data on RSV are limited in several countries within the WPR, with most data focusing on children and hospitalised patients. Further studies and surveillance, incorporating laboratory testing, are needed to determine the burden of RSV infection in the WPR countries.

Respiratory syncytial virus (RSV) is the leading cause of viral pneumonia and bronchiolitis, especially in younger children. The global estimate of RSV cases in children in 2015 was 33.1 million, with 3.2 million of these requiring hospitalisation [1]. The global estimated age-specific death rates due to RSV pneumonia in 2010 was 3.5 per 100 000 population, with RSV contributing 6.7% and 1.6% of all deaths in the 28-364 day-old and in the 1-4 year-old age groups, respectively [2]. Although most RSV studies to date have focused on children, the burden of RSV infection in adults, particularly in the older age group, is increasingly recognised [3-6]. Both asymptomatic and mild infection in adults raises the possibility that they may act as a source of paediatric infections that could be reduced through future vaccination [7].

Recently, RSV has been receiving more attention as one of the major viral respiratory pathogens causing acute lower respiratory infections in younger children. RSV epidemiological data, including incidence and prevalence, were estimated from different sources, including scientific publications and hospital reports. These data are usually limited to a specific location, community or hospital, during a specific time period, and therefore it is often difficult to conclude prevalence figures or estimate their accuracy. Several systematic reviews have tried to determine the burden of RSV infection, especially in children, in different regions in the world [1,8-10]. RSV epidemiological data are required to properly plan resource allocation and public health policies for disease control, particularly when or if a vaccine becomes available. In the future, surveillance that incorporates laboratory diagnostic testing using molecular techniques should provide information about transmission, evolution and the emergence of new RSV genotypes and strains that will help in establishing preventive measures to control RSV infection. The World Health Organization (WHO) has started piloting RSV surveillance in 14 countries since mid-2017 with the objective to obtain evidence-based data which will help in developing RSV vaccination policy [11].

The WHO Western Pacific Region (WPR), which consists of 37 countries in Asia, Oceania and the Pacific, is very diverse regarding demographics, political and socio-economic status, and includes health care systems that vary in terms of funding and resilience. The population of the WHO WPR area is approximately 1.9 billion people, which is approximately 25% of the global population [12]. There are an estimated 0.11 pneumonia episodes per child-year, with 61 900 pneumonia-related deaths annually in the WPR area [13]. The contribution of viral pathogens in acute lower respiratory tract infections in the region has, to date, mostly been provided by influenza data, as surveillance for Influenza is already conducted in 15 WPR countries [14]. However, the burden of RSV infections in the WPR region, in both adults and children is still unclear.

We reviewed RSV studies conducted in the WPR countries from January 2000 to October 2017 to investigate the epidemiological aspects of RSV: (incidence), prevalence, seasonality and hospitalisation status, and the associated molecular epidemiology data. To the best of our knowledge, this is the first review to systematically analyse RSV-related epidemiological data in the WPR countries.

## METHODS

### Search strategy

Searches were systematically carried out in the main international literature databases (MEDLINE, EMBASE,Web of Science and SCOPUS), using the search terms: “RSV” OR “respiratory syncytial virus” AND “prevalence” OR “positivity” OR “rate” OR “infection” OR “proportion” OR “frequency” in combination with each WPR country (n = 37) using the Preferred Reporting Items for Systematic Reviews and Meta-analysis (PRISMA) guidelines [15]. The date of the search was 30^th^ October 2017. The final reference list of the articles included was reviewed for additional information. We also searched the web pages of all countries’ Ministries of Health and the WHO and WPR Office websites.

### Selection criteria

Studies were included if the following criteria were fulfilled: 1) studies in humans; 2) studies in patients with acute respiratory tract infection (ARTI) or lower respiratory tract infection (LRTI) or influenza-like illness (ILI); 3) studies that reported one or more of the following: incidence, prevalence, seasonality, or molecular epidemiology (genotypes); and 4) studies published in English. Studies published or reported between January 2000 and October 2017 were included. We excluded articles if they were: 1) basic science or animal studies, 2) replication of studies, 3) nosocomial cases of RSV, or 4) case reports.

### Data extraction and analysis

Literature screening was performed by assessing title, keyword and abstract. EndNote X7.5 (Thomson Reuters) was used for bibliography management, in which duplicates were removed before the initial screening. The first screening after removing 1027 duplicates was performed by excluding the irrelevant abstracts. Full-text screening was then conducted on 370 articles and a total of 196 papers were eligible for the study. The following data from eligible studies were extracted: study location (city, country); years and duration; setting (inpatient, outpatient); participant age; number of individuals and/or respiratory samples tested; number of RSV-positive specimens; number of each type of RSV-positive specimens (RSV A and RSV B); and number of genotypes identified.

We conducted meta-analyses using the Der-Simonian and Laird method using random-effects models [16] to calculate the pool of the RSV proportions by country, inpatient/outpatient, age, and RSV detection methods. The random-effects model was used because we assumed that the true estimates vary from study to study. All analyses were performed using MedCalc software version 19.05 (MedCalc Software, Belgium).

### Primary and secondary outcomes

This systematic review included the following outcomes: (i) incidence and prevalence of RSV infections, (ii) percentage of ILIs and LRTIs being caused by RSV by demographic (age, country) and hospitalisation status (inpatients vs outpatients), and (iii) seasonality and molecular epidemiology.

## RESULTS

**Figure 1:**
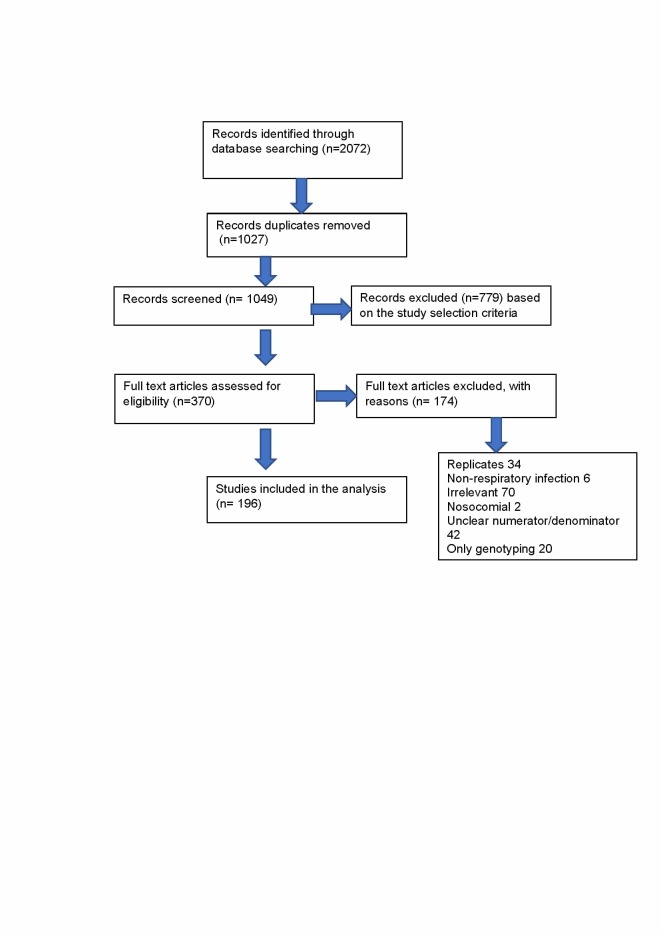
Flow diagram of studies selection. The criteria for identification of irrelevant studies adapted from previous publication [17]): (1) did not discuss respiratory tract infections or respiratory syncytial virus (RSV), (2) RSV mentioned only as background to discussion.

**Figure 2:**
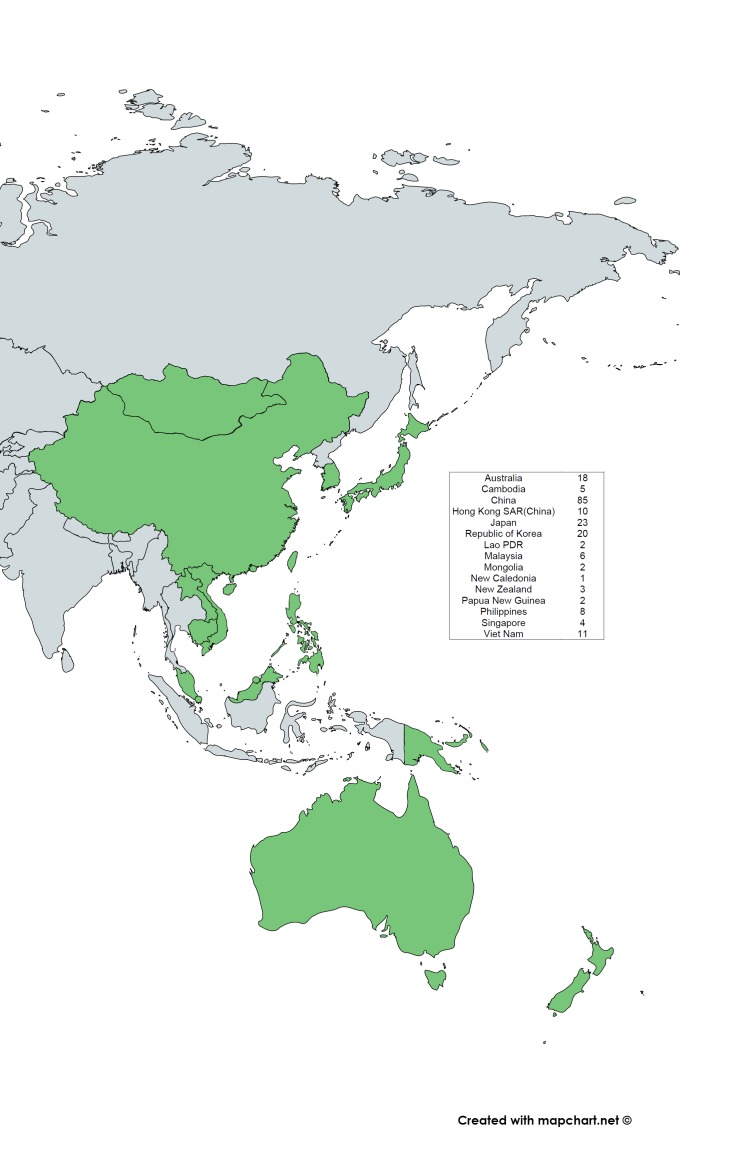
Distribution of respiratory syncytial virus (RSV) studies in 15 of the 37 Western Pacific Region countries/areas. The WPR countries/areas are in green. Map was created with mapchart.net. (Available from: https://mapchart.net/).

We identified 2072 articles, from which 196 studies were included based on our eligibility criteria (**Figure 1**), with two studies covering three WPR countries [18,19]. These included studies were only from 15 of the 37 countries listed in the WHO WPR. The map of WHO WPR countries and the distribution of the included RSV studies are shown in **Figure 2**. Of the 196 studies, 193 studies reported RSV-positivity rate, 13 studies reported the incidence, 95 studies reported seasonality and 43 studies reported RSV sub-grouping. The detailed information about the articles included in this study is provided in Table S1 in the **Online Supplementary Document****.**

### RSV positive rate

A total of 193 studies reported on the percentage of respiratory samples taken from patients with ARTI, LRTI or ILI that were positive for RSV, giving an overall positivity rate of 16.73% (95% confidence interval (CI) = 15.12%-18.4%) (**Table 1**). From the 196 studies that were included, 140 studies were conducted in children, 18 studies in adults, 38 studies covered all ages. The classification of age group (children and adult) was based on the classification used by each article. Of these studies, we then divided the age population into two categories (adult and children) by further extracting the data from the all-ages population to allow comparison of the positivity rate between the two categories. Among 868 236 children, RSV was detected in 120 137 (20.7%, 95% CI = 18.68%-22.83%); and among 54 915 adults, RSV was detected in 1003 (1.87%, 95% CI = 1.37%-2.46%). The RSV positivity rate in children is significantly higher than in adults (χ^2^ = 6535, *P* < 0.001, **Table 1**). Furthermore, the RSV positivity rate in children aged less than 5 years is higher than children older than 5 years (25.51%, 95% CI = 22.92%-28.19% vs 5.24%, 95% CI = 3.36%-7.51%, *P* < 0.001).

**Table 1:** Respiratory syncytial virus (RSV) positivity rate based on the country, age, hospitalization status and virus methods

		No. articles included	Total No. patients	No. RSV positive	% RSV positive (95% CI)
**Country**					
Australia	16		244 370	33 860	14.21 (8.06-21.76)
Cambodia	5		11 152	1002	8.80 (4.13-14.9)
China (excl. HK)	85		508 283	70 495	15.69(13.7-17.78)
Hong Kong SAR (China)	10		143 942	10 193	9.55 (3.4-18.35)
Japan	23		37 690	6533	24.74 (15.81-34.9)
Malaysia	6		76 525	3686	15.76 (4.45-32.24)
Viet Nam	11		12 273	2135	15.97 (8.68-24.96)
Philippines	8		33 242	3336	20.23 (8.17-35.97))
New Zealand	2		1601	681	50.13 (29.19-71.04)
Republic of Korea	20		80 633	6389	18.05 (13.84-22.67)
Mongolia	2		542	52	13.03 (2.36-30.4)
Singapore	4		52 156	9196	13.58 (9.64-18.06)
Lao PDR	2		675	199	26.72 (5.88-55.6)
Papua New Guinea	2		380	44	14.74 (3.99 - 30.65)
New Caledonia*	1		108	49	45.37(35.76 - 55.24)
**Total**	197†		1 203 572	147 850	16.73 (15.12-18.4)
**Age group:**					
Children	159†		868 236	120 137	20.7(18.68-22.8)
<5 y	48		184 609	42 206	25.51 (22.92-28.19)
>5 y	16		11 393	501	5.24 (3.36-7.51)
Adult	34		54 915	1003	1.87 (1.37 - 2.46)
**Hospitalisation:**					
Inpatient	155‡		968 885	118 937	18.28(16.3-20.3)
Outpatient	43‡		159 574	17 702	11.54(8.97-14.37)
**Virus detection methods§:**					
PCR	121†		307 184	40 897	16.04 (14.04 -18.15)
IF	29		459 315	42 229	17.7 (13.93-21.9)
Other	17		93 985	21 532	22.5 (17.35-28.14)
Culture Mix	29		334 925	42 381	15.65 (11.42–20.41)

RSV – respiratory syncytial virus, CI – confidence interval, y – year

*CI was not calculated.

†Two studies covered three countries.

‡One study covered three countries.

§Other-serology/Enzyme Immunoassay (EIA), Culture Mix- combination of culture and any methods, PCR – polymerase chain reaction, IF – immunofluorescence.

From the 193 studies that reported the prevalence of RSV based on the hospitalisation setting (inpatient, outpatient, or both), we classified the data into two categories, inpatients and outpatients, for analysis. There were 146 studies reporting RSV in hospitalised patients, 34 reporting RSV in outpatients, and 13 reporting RSV in both outpatients and inpatients. We also further extracted the data from these 13 articles to obtain each outpatients and inpatients data. RSV prevalence was higher in hospitalised inpatients (18.28%, 95% CI = 16.29%-20.4%) than in outpatients (11.89%, 95% CI = 9.22%-14.74%) (χ^2^ = 180, *P* < 0.001, **Table 1**). In this study, RSV prevalence of hospitalised children is higher than in outpatient cases (22.39%, 95% CI = 19.8%-25.1% vs 19.9%, 95% CI = 15.44%-24.77%, *P* < 0.001), while in outpatient adults, the positivity rate is higher than in hospitalised adults (3.11%, 95% CI = 1.36%-5.54% vs 1.43%, 95% CI = 0.79%-2.26%, *P* < 0.62).

### RSV incidence

There were 15 studies reported the RSV incidence in eight countries/areas (Australia, China, Hong Kong SAR, Mongolia, New Zealand, Philippines, Singapore, Viet Nam), mostly in hospitalised children, while the outpatient incidence from ILI patients was reported in five countries (**Table 2**). The incidence of RSV-associated hospitalisation ranged between 4.9-30.9 per 1000 child-years and varied according to age group. There was only one study that provided the incidence of RSV-associated hospitalisation in adult patients, which was 0.57 per 1000 person-years [26]. The estimated incidence of RSV-associated ILI in children ranged from 0 to 137 per 1000 child-years [19,32].

**Table 2:** Characteristic of studies which reported RSV incidence in the Western Pacific Region countries

Country	Study period	Study Design	Age	Case definition	Sample size	RSV-associated hospitalization	RSV-associated ILI	RSV Incidence	Reference
Australia	1997-2004	Retrospective case records	Children	LRTI, RSV +ve	271	22/1000 live birth	_	_	[20]
	2000-2004	Retrospective case records	<2 y	LRTI, RSV +ve	173	20.4 per 1000 children <2y	_	_	[21]
	1991-2000	Published national laboratory data	<5 y	LRTI, RSV +ve	NA	_	_	110.0-226.5 per 1000 children under 5 to 435.0-869.0 per 1000 infants	[22]
	2001-2010	Retrospective cohort	<5 y	LRTI	16,119	4.9 per 1000 CY (<5 y), 25.6 per 1000 child-years (<3 mo)	_	_	[23]
	2010-2011	Prospective cohort	6 m-10 y	ILI	82	_	0 per 1000 PY (6-11 mo), 2.1 per 1000 PY (12-23 mo), 1.1 per 1000 PY (24-35 mo), 0.95 per 1000 PY (36-59 mo), 0.25 per 1000 PY (>60 mo)	_	[19]
	2000-2012	Retrospective cohort	<5 y	LRTI	43,627	_	2.5 per 1000 PY	_	[24]
China	2011	Cross-sectional	<5 y	SARI	511	45 per 1000 children	_	_	[25]
Hong Kong	1998-2012	Cross-sectional	All ages	ARI	19,405	15.8 per 1000 PY (<5 y), 0.57 per 1000 PY (>65 y)	_	_	[26]
	2003-2006	Prospective cohort	<6 m	ARI	1,031	23.3-31.1 per 1000 children	_	_	[27]
Mongolia	2013-2015	prospective cohort study	Adult and infants under 6 mo	ILI and SARI	1260 (adult), 1340 (<6 mo)	_	pregnant women: 0.3 per 1000 PY	_	[28]
New Zealand	1995-2006	Cross-sectional	All ages	ARI	NA	_	_	Monthly annualized incidence rate was 48.4 per 100 000 pop year	[29]
Philippines	2010-2011	Prospective cohort study	6 m - 10 y	ILI	724	_	8.1 per 1000 PY (6-11 mo), 3.1 per 1000 PY (12-23 mo), 1.5 per 1000 PY (24-35 mo) 0.6 per 1000 PY (36-59 mo), 0.07 per 1000 PY (>60 mo)	_	[19]
	2012-2014	Cross-sectional	All ages	ILI and SARI	ILI: 6267, SARI:2962	1.9 per 1000 person	1.4 per 1000 person	_	[30]
Singapore	2010-2011	Prospective cohort study	6 m - 10 y	ILI	34	_	0 per 1000 PY (12-23 mo), 2.5 per 1000 PY (24-35 mo) 0.5 per 1000 PY (36-59 mo), 0.1 per 1000 PY (>60 mo)	_	[19]
Viet Nam	2007-2010	Cross-sectional	1 mth-5 y	ARI	1786	23.4 per 1000 children <2y, 9.59 per 1000 children <5y	_	_	[31]
	2009-2010	Prospective cohort study	less than 2 y	ARI	2549	8 to 30.9 per 1000 IYO	23.2 to 137 per 1000 IYO	_	[32]
	2010-2012	Cross-sectional	0-5 y	ARI	1854	5.89 to 15.47 per 1000 children <5 PY	_	_	[33]

ARI – acute respiratory infection, CY – child-years, LRTI – lower respiratory tract infection, ILI- influenza like illness, PY – person-years, SARI – severe acute respiratory infection, IYO – infant-years of observation, mo – months, y – years

### Seasonality

One hundred and nineteen of the 196 studies reported the data related with the seasonality of RSV, including the duration, the week/month of the RSV season, and the peak of RSV activity (**Table 3**). Generally, temperate countries, both in the Northern and Southern hemispheres, experienced their peak of the epidemic in the winter. In subtropical and tropical countries, the cases peaked mostly in the rainy (wet) season. Several studies from countries that have a wide latitude range, such as China and Australia, have reported different times for the peaks of RSV epidemics depending on the latitude.

**Table 3:** RSV seasonality in the WPR countries

	No articles included	Seasonality	Peak or common months
**Southern hemisphere:**			
**Australia**			
Temperate (NSW, Victoria)	4	Seasonal	Winter
Subtropical (Queensland)	1	Seasonal	Winter
Tropical (Queensland)	1	Seasonal	Rainy season
Tropical (Northern Territory)	1	Throughout the year	Rainy season
Desert (Northern Territory)	2	Throughout the year	Winter
**New Zealand**	1	Seasonal	Winter
**Northern hemisphere**			
**China**			
Central	2	Seasonal	Winter and Spring
Eastern	7	Seasonal	Winter
	7	Seasonal	Winter and Spring
	1	Seasonal	Autumn and Winter
	2	Throughout the year	
Northeastern	4	Seasonal	Winter
	1	Seasonal	Winter and Spring
Northwest	5	Seasonal	Winter and Spring
Southern	2	Throughout the year	Winter and Spring
	4	Throughout the year	Two peaks (winter to spring, summer to autumn)
	2	Seasonal	Winter
	1	Seasonal	Spring and summer
	2	Seasonal	Spring
Western	2	Seasonal	Winter
**Japan**	5	Seasonal	Winter and Autumn
	3	Seasonal	Winter
**South Korea**	8	Seasonal	Winter
	3	Seasonal	Winter and Autumn
	2	Seasonal	Winter and Spring
**Mongolia**	1	Seasonal	Winter
**Hong Kong**	3	Throughout the year	Rainy season
	3	Two peaks	Winter-Spring, Summer-Autumn
**Tropical**			
**Cambodia**	2	Throughout the year	Rainy season
**Viet Nam**	5	Throughout the year	Rainy season
	3	Seasonal	Hot and dry season
**Laos**	2	Throughout the year	Rainy season
**Malaysia**	4	Throughout the year	Rainy season
**New Caledonia**	1	Seasonal	Transition from wet to dry season
**Philippines**	3	Throughout the year	Rainy season
**Singapore**	2	Throughout the year	Rainy season

RSV – respiratory syncytial virus, WPR – Western Pacific Region

### RSV genotypes

Forty-three studies reported the RSV subgrouping (RSV A and B). From the total 13 775 RSV cases that underwent subgrouping, RSV A was identified in 8829 cases (63.43%, 95% CI = 57.34%-69.31%) and RSV B in 4272 cases (30.87%, 95% CI = 25.74%-36.25%), respectively. There were 51 RSV cases from seven studies with concurrent RSV A and B infection.

**Figure 3:**
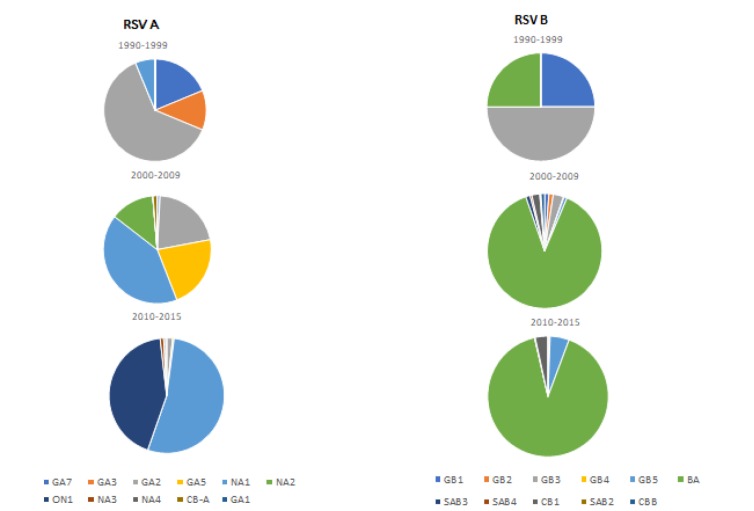
Distribution of respiratory syncytial virus (RSV) genotypes in the Western Pacific Region countries.

The distribution of RSV genotypes between 1990-2015 was reported in 34 studies from eight WPR countries. Of these 34 studies, we used 33 studies that performed genotyping based on the *G* protein gene. Eleven RSV A and RSV B genotypes were identified from 3957 RSV cases (2841 RSV A and 1116 RSV B). The distribution of each genotype is shown in **Figure 3**. NA1 and BA genotypes were the predominant genotypes reported for RSV A and B, respectively. Japan, China and Malaysia had the most complete data as they had performed multi-year molecular epidemiology studies (Table S2 in the **Online Supplementary Document**).

## DISCUSSION

Recently, RSV has received more attention as one of the major viral respiratory pathogens that cause significant mortality and morbidity, especially in younger children [2]. Several groups have determined the global burden of RSV infection in children by conducting a series of systematic reviews. The first systematic review and meta-analysis of global RSV data covering 1995-2009 estimated that, in 2005, the incidence of RSV-associated ARTI in children aged less than 5 years was 22%, resulting in 3%-9% of all deaths, most of which (90%) occurred in low- to middle-income countries [8]. Further analysis, using more recent publications and data, estimated that 28% of ARTI episodes were RSV-associated, causing 13%-22% of all ARTI mortality in children aged under 5 years in 2015 [1]. A meta-analysis of pneumonia-related data conducted by the Global Action Plan for Pneumonia and Diarrhoea (GAPPD) group found that RSV is the most common aetiology of pneumonia in children, estimating that it contributes 29% of all episodes [34]. However, a systematic review of the adult population has rarely been performed.

In this review, the RSV pooled positivity rate is 16.73% of all acute respiratory infection cases in the WPR countries. The previous estimate based just on pneumonia episodes showed a higher RSV prevalence than our result [34]. Our result shows that the RSV-positive rate in both adult and child patients is higher than a previous systematic review of studies in Africa with a pooled prevalence of RSV infection in ARTI cases of 14.6% [10]. However, our RSV pooled prevalence is lower than the prevalence from systematic review studies in Latin America and Iran, where the RSV pooled prevalence was 18.7% and 9.2%-41.5%, respectively [9,35]. The RSV positivity rate within the countries in the WPR ranged from 8.8% (Cambodia) to 50.13% (New Zealand). Our results show that the RSV prevalence in both adult and child patients in China of 15.6% (95% CI = 13.7%-17.78%) is similar to a previous systematic review where it was 18.7% (95% CI = 17.1%-20.5%) [17].

The difference in RSV prevalence may be due to differences in the number of studies conducted within different countries, with most of the studies identified being from China, Japan, the Republic of Korea and Australia. These four countries have established strong national influenza surveillance programmes, which can be easily extended to cover other respiratory viruses [36]. Many of the studies in the WPR region were from the expansion of influenza-like illness (ILI) and severe acute respiratory infection (SARI) surveillance. Japan commenced RSV surveillance in 2003 [37], while the Korean Influenza and Respiratory Virus Surveillance System (KINRESS) for major respiratory viruses commenced in 2005, with sentinel sites located nationwide [38]. China has a nationwide influenza-surveillance system, but RSV surveillance is less well-established [36,39]. In Australia there is no national RSV surveillance system, however, there is monitoring of the trends of detected infections through several laboratories [22].

The RSV surveillance pilot project conducted by the WHO is based on the Global Influenza Surveillance and Response System and aims to provide evidence-based data, including on the epidemiology and circulation of RSV, that is needed for the implementation and monitoring of any future RSV vaccine [40]. This surveillance uses the existing influenza surveillance platforms that incorporate laboratory identification and molecular characterisation to monitor circulating genotypes and identify emerging viruses. This recent effort supports the Asia Pacific Strategy for Emerging Diseases (APSED), developed by WHO WPR and South East Asian Region (SEAR) member countries, that aims to guide member countries in achieving their obligations under the International Health Regulations (IHR 2005) [41]. Furthermore, the RSV surveillance pilot project may facilitate research on respiratory viruses as proposed by the BRaVE (Battle against Respiratory Viruses) initiative in 2013 [42].

The largest population group included in our RSV study is children with acute respiratory tract infections, 159 studies, which show a prevalence higher than the adult group. Our results are similar to previous systematic studies in both developing countries [9,10] and developed countries [43,44]. The prevalence of RSV in adults is still not well-established compared with studies of adult influenza infection and the limited number of studies in adults might contribute to the lower reported prevalence in this age population. Based on our study, RSV has a considerable contribution to the WPR as an aetiology of respiratory infection, especially in children less than 5 years. Our analysis of the difference in the RSV positivity rate between children under 5 years and greater than 5 years is concordant with the results from African region [10]. As the specific prevention measure of an RSV vaccine is not yet available, other respiratory infection prevention and control measures targeted at this age group should be implemented in both developed and developing countries.

Most studies included in this review were conducted in hospital settings, with the percentage of RSV detected in inpatients being higher than in outpatients. Our results are similar to other studies where RSV prevalence was higher in hospitalised patients than in outpatients with ILI [43,45]. The RSV positivity rate in previous studies in hospitalised children varied between 3%-29% [46], which were similar to our result (22.38%). However, our study found that the RSV positivity rate in outpatient adults was higher than in hospitalised adults, which is consistent with a study conducted in the United States demonstrating a higher rate of RSV emergency department visits than RSV hospitalisation rates in adults [47].

The reported incidence of acute respiratory infection in children due to RSV in the WPR countries is broad. The incidence of RSV-associated hospitalisation in children decreased with increasing age, a finding similar to previous studies [48-50]. A systematic review of children hospitalised due to RSV found that the incidence in the western countries (Australia, USA and Europe) was higher than in Asia [49], while in our study the incidence between western countries and Asia is similar. A study from Egypt reported a higher average annual incidence of RSV in outpatients than in hospitalised inpatients [51], while in the WPR countries only one study from Viet Nam showed similar results [32]. The variability of incidence has also been discussed in other studies possibly due to location, case definition, study population and diagnostic methods [49]. Our study provides additional data regarding the RSV burden in the outpatient setting to that in western countries [50]. As there are few studies of the acute respiratory RSV infection incidence in adults in the WPR, future research should focus on this age group.

The seasonality of infectious diseases, especially viral respiratory infections, is important for planning public health responses and workforce distribution, as seen for global influenza surveillance. Our results showed similar RSV seasonality that covered several locations that were not included in the previous review [52]. In the temperate regions in both the Northern and Southern hemispheres, RSV peaked in the winter season. In tropical countries, the seasonality of RSV is not clearly defined. However, the number of cases seems to peak at different times of the year, mostly in the rainy season. In Australia, the seasonality of RSV differs by region with the northern tropical part, including Cairns and Darwin, experiencing an RSV peak in the rainy season such as seen in neighbouring tropical countries [53].

Diagnostic methods for detecting respiratory viruses were advanced recently by the development of molecular methods for determining infectious aetiology. In this analysis, 121 studies used RSV polymerase chain reaction (PCR) assays to identify the respiratory viruses present during the acute respiratory infection. The positivity rate in the studies using PCR was similar to the studies using immunofluorescence (IF), which might be related to the specificity and sensitivity of the IF techniques [10]. A previous systematic review that included only studies using PCR had a higher RSV prevalence than studies using IF and immunochromatographic methods [9,10,35. As we observed, there were reports of co-infection or co-detection of RSV with other respiratory viruses in the specimens tested, which were assumed to be due to the increased sensitivity of molecular methods [54-56].

Molecular methods have recently replaced monoclonal antibody determination of the grouping of RSV into RSV A and RSV B [57]. Recent studies analysed the distribution of RSV groups and genotypes and demonstrated that both RSV A and RSV B co-circulate in the same epidemic period with shifting predominance of the groups [58-61]. Further molecular characterisation studies identified various genotypes within each group, with each genotype able to cluster temporally, locally or circulate at different times and different global locations [58]. The molecular epidemiology studies conducted in the WHO WPR countries demonstrated the co-circulation of RSV A and RSV B with shifting dominance occurring between groups and changes to the diversity of the genotypes. RSV A was more dominant than RSV B in the WPR countries during the past two decades. This finding is concordant with studies conducted in other parts of the world [59-62].

Several studies in the WPR countries included sequencing to differentiate specific genotypes and examine their circulation, estimate pathways of transmission, and detect new and emerging genotypes. Japan, China, and Malaysia had relatively comprehensive molecular data over more extended periods. However, these molecular studies from large countries are often limited to a few sentinel sites and therefore may not be representative of the whole country’s disease burden. RSV genotype circulation in the WPR countries followed a similar pattern to global circulation [63], as can be observed by the emergence of the new genotypes, BA (RSV B) and ON1 (RSV A). The RSV B BA genotype, first detected in specimens from Buenos Aires in 1999 [64], was first identified in the WPR from Malaysia’s specimens in 1999 [65]. The BA genotype then further identified in several countries in the WPR region and became the dominant RSV B genotype. The distribution of RSV A ON1 genotype in the WPR countries also followed a similar pattern as in other global regions. The ON1 genotype was first identified in specimens from Ontario in 2010 [66] and emerged in 2011 in the WPR countries; in China [67], Malaysia [65] and Republic of Korea [68]. In the Southern hemisphere, the molecular epidemiology of RSV is not well-established as studies from Australia and New Zealand are limited. As an RSV vaccine is currently under development, data on the distribution of RSV strains will be important both for the composition of the vaccine and for post-vaccine implementation, particularly with regard to any immune selection pressure exerted by the vaccine.

Our study has some limitations. First, there were variations in the case definitions, study design, and pathogen detection methods used in these included studies. We had difficulty in classifying the children’s age groups, as each countries’ studies had differing age ranges. Therefore, the interpretation of the results has been cautious due to the difficulties in comparing some of these studies. In the future, it would be desirable to define the age-ranges of the children and adults when conducting epidemiology studies globally. Second, RSV is not the main focus of several studies, as most studies focused on finding other possible causes of respiratory infections. Third, the advances in PCR technology with increased sensitivity, which incorporate the detection of multiple respiratory viruses, allows for the inclusion of RSV, where it may not be the active infection. Therefore, as in the previous systematic review (10), we should consider the presence of viral co-infections in our future results. Lastly, the amount of data available per country is variable, with only half of the WPR countries having reported RSV and multiple studies from certain countries, which may bias the data, Despite this, our study has attempted to summarise data on the epidemiology of RSV in both adults and children in the WPR.

## CONCLUSION

This study suggests that the RSV has considerable prevalence in the WPR countries, although the RSV data are limited as several countries have focused more on infections in children and hospitalised patients. The seasonality and the molecular epidemiology of RSV among WPR countries are similar to and reflect the global pattern. Further studies and surveillance incorporating molecular laboratory typing in adults and out-patients are needed to determine the overall burden of RSV infection in the WPR countries.

## Additional material

Online Supplementary Document

## References

[1] Shi T McAllister DA O’Brien KL Simoes EAF Madhi SA Gessner BD Global, regional, and national disease burden estimates of acute lower respiratory infections due to respiratory syncytial virus in young children in 2015: a systematic review and modelling study. Lancet. 2017;390:946-58. 10.1016/S0140-6736(17)30938-8 28689664 PMC5592248

[2] Lozano R Naghavi M Foreman K Lim S Shibuya K Aboyans V Global and regional mortality from 235 causes of death for 20 age groups in 1990 and 2010: a systematic analysis for the Global Burden of Disease Study 2010. Lancet. 2012;380:2095-128. 10.1016/S0140-6736(12)61728-0 23245604 PMC10790329

[3] Falsey AR Cunningham CK Barker WH Kouides RW Yuen JB Menegus M Respiratory syncytial virus and influenza A infections in the hospitalized elderly. J Infect Dis. 1995;172:389-94. 10.1093/infdis/172.2.389 7622882

[4] Malosh RE Martin ET Callear AP Petrie JG Lauring A Lamerato L Respiratory syncytial virus hospitalization in middle-aged and older adults. J Clin Virol. 2017;96:37-43. 10.1016/j.jcv.2017.09.001 28942341 PMC5889293

[5] Binder W Thorsen J Borczuk P RSV in adult ED patients: Do emergency providers consider RSV as an admission diagnosis? Am J Emerg Med. 2017;35:1162-5. 10.1016/j.ajem.2017.06.022 28633906

[6] Pastula ST Hackett J Coalson J Jiang X Villafana T Ambrose C Hospitalizations for Respiratory Syncytial Virus Among Adults in the United States, 1997-2012. Open Forum infect Dis. 2017;9:ofw270. 10.1093/ofid/ofw270 PMC5414053 28480262

[7] Hall CB Long CE Schnabel KC Respiratory syncytial virus infections in previously healthy working adults. Clin Infect Dis. 2001;33:792-6. 10.1086/322657 11512084

[8] Nair H Nokes DJ Gessner BD Dherani M Madhi SA Singleton RJ Global burden of acute lower respiratory infections due to respiratory syncytial virus in young children: a systematic review and meta-analysis. Lancet. 2010;375:1545-55. 10.1016/S0140-6736(10)60206-1 20399493 PMC2864404

[9] Bardach A Rey-Ares L Cafferata ML Cormick G Romano M Ruvinsky S Systematic review and meta-analysis of respiratory syncytial virus infection epidemiology in Latin America. Rev Med Virol. 2014;24:76-89. 10.1002/rmv.1775 24757727

[10] Kenmoe S Bigna JJ Well EA Simo FBN Penlap VB Vabret A Prevalence of human respiratory syncytial virus infection in people with acute respiratory tract infections in Africa: A systematic review and meta-analysis. Influenza Other Respir Viruses. 2018;12:793-803. 10.1111/irv.12584 29908103 PMC6185896

[11] World Health Organization. Respiratory Syncytial Virus (RSV). Available: http://www.who.int/influenza/rsv/en. Accessed: 23 February 2018.

[12] World Health Organization. WHO in the Western Pacific. Available: http://www.wpro.who.int/about/in_brief/en/. Accessed: 23 February 2018.

[13] Rudan I Boschi-Pinto C Biloglav Z Mulholland K Campbell H Epidemiology and etiology of childhood pneumonia. Bull World Health Organ. 2008;86:408-16. 10.2471/BLT.07.048769 18545744 PMC2647437

[14] Members of the WHO Western Pacific Region Global Influenza Surveillance and Response System Epidemiological and virological characteristics of seasonal influenza in the Western Pacific Region of the World Health Organization, 2011-2015. Western Pac Surveill Response J. 2017;8:40-9. 10.5365/wpsar.2017.8.1.004 28409059 PMC5375099

[15] Moher D Liberati A Tetzlaff J Altman DG Preferred reporting items for systematic reviews and meta-analyses: the PRISMA statement. J Clin Epidemiol. 2009;62:1006-12. 10.1016/j.jclinepi.2009.06.005 19631508

[16] DerSimonian R Laird N Meta-analysis in clinical trials. Control Clin Trials. 1986;7:177-88. 10.1016/0197-2456(86)90046-2 3802833

[17] Zhang Y Yuan L Zhang Y Zhang X Zheng M Kyaw MH Burden of respiratory syncytial virus infections in China: Systematic review and meta-analysis. J Glob Health. 2015;5:020417. 10.7189/jogh.05.020417 26682049 PMC4676581

[18] Benet T Sanchez Picot V Messaoudi M Chou M Eap T Wang J Microorganisms Associated with Pneumonia in Children <5 Years of Age in Developing and Emerging Countries: The GABRIEL Pneumonia Multicenter, Prospective, Case-Control Study. Clin Infect Dis. 2017;65:604-12. 10.1093/cid/cix378 28605562 PMC7108107

[19] Nolan T Borja-Tabora C Lopez P Weckx L Ulloa-Gutierrez R Lazcano-Ponce E Prevalence and incidence of respiratory syncytial virus and other respiratory viral infections in children aged 6 months to 10 years with influenza-like illness enrolled in a randomized trial. Clin Infect Dis. 2015;60:e80-9. 10.1093/cid/civ065 25673560 PMC4429758

[20] Reeve CA Whitehall JS Buettner PG Norton R Reeve DM Francis F Predicting respiratory syncytial virus hospitalisation in Australian children. J Paediatr Child Health. 2006;42:248-52. 10.1111/j.1440-1754.2006.00849.x 16712553

[21] Dede A Isaacs D Torzillo PJ Wakerman J Roseby R Fahy R Respiratory syncytial virus infections in Central Australia. J Paediatr Child Health. 2010;46:35-9. 10.1111/j.1440-1754.2009.01614.x 19943864

[22] Ranmuthugala G Brown L Lidbury BA Respiratory syncytial virus–the unrecognised cause of health and economic burden among young children in Australia. Commun Dis Intell Q Rep. 2011;35:177-84. 22010512 10.33321/cdi.2011.35.15

[23] Homaira N Oei JL Mallitt KA Abdel-Latif ME Hilder L Bajuk B High burden of RSV hospitalization in very young children: A data linkage study. Epidemiol Infect. 2016;144:1612-21. 10.1017/S0950268815003015 26626237 PMC9150613

[24] Lim FJ Blyth CC Fathima P de Klerk N Moore HC Record linkage study of the pathogen-specific burden of respiratory viruses in children. Influenza Other Respir Viruses. 2017;11:502-10. 10.1111/irv.12508 28991397 PMC5705691

[25] Huo X Fang B Liu L Yu H Chen H Zheng J Clinical and epidemiologic characteristics of respiratory syncytial virus infection among children aged <5 years, Jingzhou city, China, 2011. J Infect Dis. 2013;208 SUPPL. 3:S184-8. 10.1093/infdis/jit518 24265477

[26] Chan PKS Tam WWS Lee TC Hon KL Lee N Chan MCW Hospitalization incidence, mortality, and seasonality of common respiratory viruses over a period of 15 years in a developed subtropical city. Medicine (Baltimore). 2015;94:e2024. 10.1097/MD.0000000000002024 26579810 PMC4652819

[27] Chiu SS Chan KH Chen H Young BW Lim W Wong WH Virologically confirmed population-based burden of hospitalization caused by respiratory syncytial virus, adenovirus, and parainfluenza viruses in children in Hong Kong. Pediatr Infect Dis J. 2010;29:1088-92. 10.1097/INF.0b013e3181e9de24 20622713

[28] Chaw L Kamigaki T Burmaa A Urtnasan C Od I Nyamaa G Burden of Influenza and Respiratory Syncytial Virus Infection in Pregnant Women and Infants Under 6 Months in Mongolia: A Prospective Cohort Study. PLoS One. 2016;11:e0148421. 10.1371/journal.pone.0148421 26849042 PMC4746066

[29] Murdoch DR Jennings LC Association of respiratory virus activity and environmental factors with the incidence of invasive pneumococcal disease. J Infect. 2009;58:37-46. 10.1016/j.jinf.2008.10.011 19042025

[30] Kamigaki T Aldey PP Mercado ES Tan AG Javier JB Lupisan SP Estimates of influenza and respiratory syncytial virus incidences with fraction modeling approach in Baguio City, the Philippines, 2012-2014. Influenza Other Respir Viruses. 2017;11:311-8. 10.1111/irv.12453 28371393 PMC5485869

[31] Yoshida LM Suzuki M Nguyen HA Le MN Vu TD Yoshino H Respiratory syncytial virus: Co-infection and paediatric lower respiratory tract infections. Eur Respir J. 2013;42:461-9. 10.1183/09031936.00101812 23645407

[32] Anders KL Nguyen HL Nguyen NM Van Thuy NT Hong Van NT Hieu NT Epidemiology and virology of acute respiratory infections during the first year of life: A birth cohort study in Vietnam. Pediatr Infect Dis J. 2015;34:361-70. 10.1097/INF.0000000000000643 25674708 PMC4418783

[33] Yoshihara S Kusuda S Mochizuki H Okada K Nishima S Simoes EAF Effect of palivizumab prophylaxis on subsequent recurrent wheezing in preterm infants. Pediatrics. 2013;132:811-8. 10.1542/peds.2013-0982 24127479

[34] Rudan I O’Brien KL Nair H Liu L Theodoratou E Qazi S Epidemiology and etiology of childhood pneumonia in 2010: estimates of incidence, severe morbidity, mortality, underlying risk factors and causative pathogens for 192 countries. J Glob Health. 2013;3:010401. 23826505 10.7189/jogh.03.010401 PMC3700032

[35] Salimi V Tavakoli-Yaraki M Yavarian J Bont L Mokhtari-Azad T Prevalence of human respiratory syncytial virus circulating in Iran. J Infect Public Health. 2016;9:125-35. 10.1016/j.jiph.2015.05.005 26143136

[36] Jennings L Huang QS Barr I Lee PI Kim WJ Buchy P Literature review of the epidemiology of influenza B disease in 15 countries in the Asia-Pacific region. Influenza Other Respir Viruses. 2018;12:383-411. 10.1111/irv.12522 29127742 PMC5907823

[37] Kanou K Arima Y Kinoshita H Ito H Okuno H Saito N Respiratory Syncytial Virus Surveillance System in Japan: Assessment of Recent Trends, 2008-2015. Jpn J Infect Dis. 2018;71:250-5. 10.7883/yoken.JJID.2017.261 29709974

[38] Kim JM Jung HD Cheong HM Lee A Lee NJ Chu H Nation-wide surveillance of human acute respiratory virus infections between 2013 and 2015 in Korea. J Med Virol. 2018;90:1177-83. 10.1002/jmv.25069 29488229 PMC7166751

[39] Yu J Liu C Xiao Y Xiang Z Zhou H Chen L Respiratory Syncytial Virus Seasonality, Beijing, China, 2007-2015. Emerg Infect Dis. 2019;25:1127-35. 10.3201/eid2506.180532 31107230 PMC6537707

[40] World Health Organization. WHO Technical Meeting on Piloting RSV Surveillance based on the Global Influenza Surveillance and Response System. Available: http://www.who.int/influenza/resources/publications/Technical_Meeting_RSV_Pilot/en. Accessed February 23, 2018.

[41] World Health Organization. Asia Pacific Strategy for Emerging Diseases. 2010. Available: http://www.wpro.who.int/emerging_diseases/documents/docs/asped_2010.pdf. Accessed: 23 February 2018.

[42] World Health Organization. Research need for the Battle against Respiratory Viruses (BRaVe). Available: http://www.who.int/influenza/patient_care/clinical/BRaVe_Research_Agenda_2013.pdf. Accessed: 23 February 2018.

[43] Alimi Y Lim WS Lansbury L Leonardi-Bee J Nguyen-Van-Tam JS Systematic review of respiratory viral pathogens identified in adults with community-acquired pneumonia in Europe. J Clin Virol. 2017;95:26-35. 10.1016/j.jcv.2017.07.019 28837859 PMC7185624

[44] Meerhoff TJ Fleming D Smith A Mosnier A van Gageldonk-Lafeber AB Paget WJ Surveillance recommendations based on an exploratory analysis of respiratory syncytial virus reports derived from the European Influenza Surveillance System. BMC Infect Dis. 2006;6:128. 10.1186/1471-2334-6-128 16899110 PMC1560143

[45] Druce J Tran T Kelly H Kaye M Chibo D Kostecki R Laboratory diagnosis and surveillance of human respiratory viruses by PCR in Victoria, Australia, 2002-2003. J Med Virol. 2005;75:122-9. 10.1002/jmv.20246 15543580 PMC7166941

[46] Pavia AT Viral infections of the lower respiratory tract: old viruses, new viruses, and the role of diagnosis. Clin Infect Dis. 2011;52 Suppl 4:S284-9. 10.1093/cid/cir043 21460286 PMC3106235

[47] Widmer K Griffin MR Zhu Y Williams JV Talbot HK Respiratory syncytial virus- and human metapneumovirus-associated emergency department and hospital burden in adults. Influenza Other Respir Viruses. 2014;8:347-52. 10.1111/irv.12234 24512531 PMC3984605

[48] Emukule GO Khagayi S McMorrow ML Ochola R Otieno N Widdowson MA The burden of influenza and RSV among inpatients and outpatients in rural western Kenya, 2009-2012. PLoS One. 2014;9:e105543. 10.1371/journal.pone.0105543 25133576 PMC4136876

[49] Stein RT Bont LJ Zar H Polack FP Park C Claxton A Respiratory syncytial virus hospitalization and mortality: Systematic review and meta-analysis. Pediatr Pulmonol. 2017;52:556-69. 10.1002/ppul.23570 27740723 PMC5396299

[50] Bont L Checchia PA Fauroux B Figueras-Aloy J Manzoni P Paes B Defining the Epidemiology and Burden of Severe Respiratory Syncytial Virus Infection Among Infants and Children in Western Countries. Infect Dis Ther. 2016;5:271-98. 10.1007/s40121-016-0123-0 27480325 PMC5019979

[51] Rowlinson E Dueger E Taylor T Mansour A Van Beneden C Abukela M Incidence and clinical features of respiratory syncytial virus infections in a population-based surveillance site in the Nile Delta Region. J Infect Dis. 2013;208 Suppl 3:S189-96. 10.1093/infdis/jit457 24265478

[52] Bloom-Feshbach K Alonso WJ Charu V Tamerius J Simonsen L Miller MA Latitudinal variations in seasonal activity of influenza and respiratory syncytial virus (RSV): a global comparative review. PLoS One. 2013;8:e54445. 10.1371/journal.pone.0054445 23457451 PMC3573019

[53] Hogan AB Anderssen RS Davis S Moore HC Lim FJ Fathima P Time series analysis of RSV and bronchiolitis seasonality in temperate and tropical Western Australia. Epidemics. 2016;16:49-55. 10.1016/j.epidem.2016.05.001 27294794

[54] Cilla G Onate E Perez-Yarza EG Montes M Vicente D Perez-Trallero E Viruses in community-acquired pneumonia in children aged less than 3 years old: High rate of viral coinfection. J Med Virol. 2008;80:1843-9. 10.1002/jmv.21271 18712820 PMC7166914

[55] Templeton KE Scheltinga SA van den Eeden WC Graffelman AW van den Broek PJ Claas EC Improved diagnosis of the etiology of community-acquired pneumonia with real-time polymerase chain reaction. Clin Infect Dis. 2005;41:345-51. 10.1086/431588 16007532 PMC7107904

[56] Mahony JB Detection of respiratory viruses by molecular methods. Clin Microbiol Rev. 2008;21:716-47. 10.1128/CMR.00037-07 18854489 PMC2570148

[57] Sullender WM Respiratory syncytial virus genetic and antigenic diversity. Clin Microbiol Rev. 2000;13:1-15. 10.1128/CMR.13.1.1 10627488 PMC88930

[58] Cane PA Molecular epidemiology of respiratory syncytial virus. Rev Med Virol. 2001;11:103-16. 10.1002/rmv.305 11262529

[59] Peret TC Hall CB Hammond GW Piedra PA Storch GA Sullender WM Circulation patterns of group A and B human respiratory syncytial virus genotypes in 5 communities in North America. J Infect Dis. 2000;181:1891-6. 10.1086/315508 10837167

[60] Houspie L Lemey P Keyaerts E Reijmen E Vergote V Vankeerberghen A Circulation of HRSV in Belgium: from multiple genotype circulation to prolonged circulation of predominant genotypes. PLoS One. 2013;8:e60416. 10.1371/journal.pone.0060416 23577109 PMC3618235

[61] Esposito S Piralla A Zampiero A Bianchini S Di Pietro G Scala A Characteristics and their clinical relevance of respiratory syncytial virus types and genotypes circulating in northern Italy in five consecutive winter seasons. PLoS One. 2015;10:e0129369. 10.1371/journal.pone.0129369 26047100 PMC4457818

[62] Arbiza J Delfraro A Frabasile S Molecular epidemiology of human respiratory syncytial virus in Uruguay: 1985-2001–a review. Mem Inst Oswaldo Cruz. 2005;100:221-30. 10.1590/S0074-02762005000300001 16113858

[63] Pangesti KNA Abd El Ghany M Walsh MG Kesson AM Hill-Cawthorne GA Molecular epidemiology of respiratory syncytial virus. Rev Med Virol. 2018;•••:28. 29377415 10.1002/rmv.1968

[64] Trento A Galiano M Videla C Carballal G Garcia-Barreno B Melero JA Major changes in the G protein of human respiratory syncytial virus isolates introduced by a duplication of 60 nucleotides. J Gen Virol. 2003;84:3115-20. 10.1099/vir.0.19357-0 14573817

[65] Khor CS Sam IC Hooi PS Chan YF Displacement of predominant respiratory syncytial virus genotypes in Malaysia between 1989 and 2011. Infect Genet Evol. 2013;14:357-60. 10.1016/j.meegid.2012.12.017 23305888

[66] Eshaghi A Duvvuri VR Lai R Nadarajah JT Li A Patel SN Genetic variability of human respiratory syncytial virus A strains circulating in Ontario: a novel genotype with a 72 nucleotide G gene duplication. PLoS One. 2012;7:e32807. 10.1371/journal.pone.0032807 22470426 PMC3314658

[67] Cui G Qian Y Zhu R Deng J Zhao L Sun Y Emerging human respiratory syncytial virus genotype ON1 found in infants with pneumonia in Beijing, China. Emerg Microbes Infect. 2013;2:e22. 10.1038/emi.2013.19 26038462 PMC3639546

[68] Kim YJ Kim DW Lee WJ Yun MR Lee HY Lee HS Rapid replacement of human respiratory syncytial virus A with the ON1 genotype having 72 nucleotide duplication in G gene. Infect Genet Evol. 2014;26:103-12. 10.1016/j.meegid.2014.05.007 24820343 PMC7106136

