# Supplementary material for: Respiratory syncytial virus in the Western Pacific Region: a systematic review and meta-analysis
Source: J Glob Health. 2019 Dec 16;9(2):020431. doi: 10.7189/jogh.09.020431 (PMC6925967; doi:10.7189/jogh.09.020431)
Supplement: Online Supplementary Document [file jogh-09-020431-s001.pdf]

Supplementary Table 1. List of the article included in this study

No	Author	Country	Total specimen	Age group	Setting	Detection methods
1	Anders,2015	Vietnam	566	Children	outpatient	PCR
2	Arden,2006	Australia	315	All ages	inpatient	PCR,IF
3	Arnott,2011	Cambodia	7829	All ages	both	PCR
4	Baek YH,2012	Korea	965	Children	inpatient	PCR
5	Benet,2017	China	39	Children	inpatient	PCR
	Benet,2017	Mongolia	108	Children	inpatient	PCR
	Bennet,2017	Cambodia	179	Children	inpatient	PCR
6	Bolisetty,2005	Australia	167	Children	inpatient	PCR,IF,Culture
7	Buecher C, 2010	Cambodia	234	All ages	outpatient	PCR
8	Cai XY,2014	China	1980	Children	inpatient	PCR
9	Chan DCW,2007	HongKong	561	Children	inpatient	IF,vulture
10	Chan PKS,2015	HongKong	19405	All ages	inpatient	IF
11	Chan PW,2002	Malaysia	5691	Children	inpatient	Virus Culture, Directigen
12	Charles PG,2008	Australia	885	Adult	inpatient	PCR
13	Chaw L,2016	Mongolia	434	All ages	both	IF
14	Chen K,2015	China	1204	Children	inpatient	Serology
15	Chen X,2010	China	878	Children	inpatient	PCR
16	Chen Y,2016	China	4130	Children	inpatient	PCR
17	Chen Z,2014	China	42104	Children	inpatient	PCR
18	Chen ZR,2014	China	998	Children	inpatient	PCR
19	Chidlow,2012	Papua New Guinea	80	Children	outpatient	PCR
20	Chiu SS,2010	HongKong	1031	Children	inpatient	IF,Culture
21	Cho HJ,2013	Korea	108	Children	inpatient	PCR
22	Choi EH,2006	Korea	515	Children	inpatient	PCR
23	Chow A, 2006	Singapore	51370	All ages	both	IF and Virus

24	Chun JK,2009	Korea	296	Children	inpatient	PCR
25	Chung JY,2006	Korea	381	Children	inpatient	IF
26	Chung JY,2007	Korea	233	Children	inpatient	IF
27	Cui B,2015	China	1074	Children	outpatient	PCR
28	Cui G,2013	China	19942	Children	inpatient	IF
29	Dang JL,2017	China	411	Children	inpatient	PCR
30	Dapat IC,2010	Japan	1525	Children	outpatient	PCR
31	Dede,2010	Australia	NA	Children	inpatient	IF
32	Do AHL, 2011	Vietnam	309	Children	inpatient	PCR
33	Do La,2016	Vietnam	632	Children	inpatient	PCR
34	Dong W,2016	China	2819	Children	inpatient	PCR
35	Etemadi,2013	Malaysia	165	Children	inpatient	PCR
36	Fagan,2017	Australia	4305	All ages	inpatient	PCR, Culture
37	Fan,2017	China	238	Children	inpatient	PCR, IF
38	Fu YF, 2015	China	1970	All ages	outpatient	PCR
39	Feng L,2014	China	28369	All ages	inpatient	PCR
40	Fuller DG,2005	Australia	3955	Children	inpatient	Culture, IF
41	Grimwood,2008	New Zealand	230	Children	inpatient	IF
42	Guerrier,2013	Cambodia	1006	Children	inpatient	PCR
43	Han TH,2007	Korea	827	Children	inpatient	IF
44	Hara M,2014	Japan	495	Children	outpatient	PCR,Culture
45	Hara, 2008	Japan	379	Children	outpatient	EIA
46	Harada,2013	Japan	371	Children	inpatient	Immunochromatography
47	He Y,2014	China	2025	Children	inpatient	PCR
48	Homaira,2016	Australia	68913	Children	inpatient	PCR,IF
49	Hu P,2017	China	1827	Children	inpatient	PCR
50	Huang G,2013	China	279	Children	inpatient	PCR
51	Hui Ds, 2009	HongKong	259	Adult	outpatient	PCR
52	Huo X,2012	China	486	All ages	outpatient	PCR
53	Huo X,2013	China	511	Children	inpatient	PCR

54	Ikematsu,2012	Japan	401	Adult	outpatient	PCR
55	Jacoby P,2017	Australia	87981	Children	inpatient	PCR, IF,Culture
56	Ji Wei, 2010	china	7789	Children	inpatient	IF
57	Jin Y,2009	China	406	Children	inpatient	PCR
58	Jin Y,2012	China	813	Children	inpatient	PCR
59	Ju X, 2014	China	1046	All ages	outpatient	PCR
60	Kadji, 2016	Philippines	440	Children	inpatient	PCR
61	Kaida, 2014	Japan	1044	Children	outpatient	PCR
62	Kamigaki,2016	Philippines	7141	All ages	outpatient	PCR
63	Kamigaki,2017	Philippines	9229	All ages	both	PCR
64	Kaneko,2002	Japan	535	Children	inpatient	EIA, Serology
65	Khor CS,2012	Malaysia	10,269	Children	inpatient	IF, culture
66	Kim CK, 2010	Korea	1008	Children	inpatient	PCR, IF
67	Kim KY,2017	Korea	504	Children	inpatient	PCR
68	Kim MR, 2000	Korea	1389	Children	inpatient	IF,Culture
69	Kim YJ,2014	Korea	36404	All ages	inpatient	PCR
70	Kim YK,2008	Korea	400	Children	inpatient	Directigen, Culture
71	Ko DH,2017	Korea	3467	All ages	inpatient	PCR
72	Ko,2007	HongKong	505	Adult	inpatient	culture
73	Koh C, 2017	Singapore	237	Children	inpatient	IF
74	Kono,2014	Papua New Guinea	300	All ages	outpatient	PCR
75	Kushibuchi, 2013	Japan	739	Children	inpatient	PCR, Culture
76	Kusuda, 2011	Japan	8163	Children	inpatient	NA
77	Lambert SB,2007	Australia	483	Children	outpatient	PCR
78	Lazzaro, 2007	Australia	73	Children	inpatient	Culture,IF
79	Lee WK,2006	HongKong	185	Children	inpatient	serology, other
80	Li H,2013	China	924	All ages	outpatient	PCR
81	Liao H,2015	China	70	Adult	outpatient	PCR
82	Liao X,2015	China	12502	All ages	inpatient	PCR

83	Lim, 2017	Australia	43627	Children	inpatient	PCR, Culture
84	Lim,2017	Australia	2356	Children	inpatient	PCR, Culture
85	Liu C,2015	China	3356	All ages	inpatient	PCR
86	Liu J,2014	China	2407	Children	outpatient	PCR
87	Liu J,2015	China	39756	Children	inpatient	IF
88	Liu T,2015	China	607	All ages	inpatient	PCR
89	Liu W,2016	China	5483	Children	inpatient	PCR
90	Liu Wk,2014	China	4242	Children	inpatient	PCR
91	Liu Y,2016	China	4449	Children	inpatient	Culture
92	Lu G,2013	China	1028	Children	inpatient	PCR
93	Lu L,2015	China	1803	Children	inpatient	PCR
94	Lu R,2012	China	981	Adult	inpatient	PCR
95	Lu Y,2013	China	596	Adult	inpatient	PCR
96	Lu Y,2013	China	720	Children	both	PCR
97	Ma HM,2013	HongKong	475	Adult	inpatient	Culture
98	Malasao,2015	Philippines	1505	Children	inpatient	PCR
99	Mermond,2012	New Caledonia	108	Children	inpatient	PCR
100	Miyaji,2013	Japan	214	Children	inpatient	PCR
101	Mizuta,2013	Japan	13325	All Ages	inpatient	Culture
102	Moore,2012	Australia	8166	Children	inpatient	PCR,IFA,Culture
103	Moriyama,2010	Japan	402	Children	inpatient	PCR
104	Murdoch	New Zealand	NA	All Ages	both	IFA
105	Nathan AM,2014	Malaysia	604	Children	inpatient	IFA
106	Ng KF,2017	Malaysia	59291	Children	inpatient	DFA
107	Nguyen DNT,2016	Vietnam	480	All ages	outpatient	PCR
108	Nguyen, 2017	Lao	383	Children	inpatient	PCR
109	Nguyen, 2017	Vietnam	975	All ages	inpatient	PCR
110	Nishimura,2005	Japan	892	Children	outpatient	EIA
111	Noh,2013	Korea	1983	Adult	outpatient	PCR
112	Nolan et al,2015	Australia	111	Children	outpatient	PCR

	Nolan et al.2015	Singapore	49	Children	outpatient	PCR
	Nolan et al.2015	Philippines	1045	Children	outpatient	PCR
113	O'Grady,2017	Australia	817	Children	inpatient	PCR
114	Ohno,2013	Philippines	2150	Children	inpatient	PCR
115	Park E,2017	Korea	4028	All ages	outpatient	PCR
116	Park JY,2016	Korea	291	Adult	outpatient	PCR
117	Park HW,2012	Korea	175	Children	inpatient	Directigen, Culture
118	Park K, 2017	Korea	3531	All ages	inpatient	PCR
119	Paynter,2013	Philippines	10913	Children	inpatient	PCR,culture
120	Qin X,2013	China	921	Children	inpatient	PCR
121	Qu JX,2015	China	1013	Adult	inpatient	PCR
122	Rahman MM,2014	Malaysia	505	All ages	inpatient	PCR, Culture, IF
123	Ranmuthugala,2011	Australia	NA	Children	inpatient	NA
124	Reeve, 2006	Australia	12171	Children	inpatient	IF,Culture
125	Ren L, 2009	China	5808	Adult	outpatient	PCR
126	Ren,2015	China	3167	Children	inpatient	PCR,Culture
127	Saraya, 2017	Japan	106	Adult	Both	PCR
128	Sato, 2005	Japan	499	Children	outpatient	PCR
129	Sentilhes,2013	Lao	292	All ages	inpatient	PCR
130	Seo KH, 2017	Korea	322	Adult	inpatient	PCR
131	Seo YB,2014	Korea	23806	All ages	inpatient	Culture, IF
132	Shi,2015	China	3978	All ages	inpatient	PCR
133	Shobugawa,2009	Japan	1103	Children	outpatient	PCR
134	Song, 2017	China	4246	All ages	Both	PCR
135	Sugaya, 2000	Japan	1959	Children	inpatient	EIA
136	Sun H, 2016	China	709	Children	inpatient	PCR
137	Sung, 2009	Hong Kong	475	Children	inpatient	PCR
138	Suzuki, 2012	Philippines	819	Children	inpatient	PCR
139	Takeyama,2014	Japan	412	Children	inpatient	PCR
140	Tan BH,2009	Singapore	500	Children	inpatient	PCR

141	Tan W,2016	China	700	Children	inpatient	PCR
142	Tang LF, 2008	China	34885	Children	inpatient	Culture, IF
143	Tian DD,2017	China	36500	Children	outpatient	PCR,IF
144	Tran DN, 2013	Vietnam	1082	Children	inpatient	PCR
145	Tran TD,2012	Vietnam	2144	All ages	both	PCR
146	Trenholme AA, 2017	New Zealand	1371	Children	inpatient	PCR
147	Tsung, 2010	Hong Kong	475	Children	inpatient	IF
148	Tsukagoshi, 2013	Japan	1113	Children	outpatient	PCR,Culture
149	Tuan TA,2015	Vietnam	1439	Children	inpatient	PCR
150	Vong S, 2013	Cambodia	1904	Children	inpatient	PCR
151	Wang D, 2016	China	3662	Children	inpatient	PCR
152	Wang H,2016	China	30443	Children	outpatient	IF
153	Wang W,2010	China	817	Children	inpatient	PCR
154	Wang Y, 2013	China	6655	Children	inpatient	IF
155	Wang Y,2015	China	674	Children	inpatient	IF
156	Watson, 2006	Australia	10,045	All ages	inpatient	IF,Culture
157	Wei L, 2015	China	3181	Children	inpatient	PCR
158	Wertheim HFL, 2015	Vietnam	800	All ages	inpatient	PCR
159	Wu Z, 2014	China	10435	Children	inpatient	Serology
160	Xia Q,2014	China	1800	Children	inpatient	PCR, Culture
161	Xiang Z, 2013	China	9871	Adult	inpatient	PCR
162	Xiao Q, 2015	China	1742	Children	inpatient	PCR
163	Xie M, 2017	China	8031	Children	inpatient	IF
164	Xu L, 2012	China	3460	All ages	inpatient	PCR
165	Yamaguchi M, 2011	Japan	1560	Children	outpatient	PCR
166	Yan XL,2017	China	387	Children	inpatient	PCR
167	Yan Y,2017	China	6196	Children	inpatient	IF
168	Yang L,2015	HongKong	120571	All ages	inpatient	IF
169	Yasuno T, 2008	Japan	54	Children	inpatient	EIA
170	Ye C, 2017	China	967	Adult	both	PCR

171	Ye Q,2016	China	36500	Children	outpatient	IF
172	Yoshida A,2012	Japan	709	Children	inpatient	PCR,Culture
173	Yoshida LM, 2013	Vietnam	1992	Children	inpatient	PCR
174	Yoshihara, 2016	Vietnam	1854	Children	inpatient	PCR
175	Yu X, 2012	China	416	Adult	inpatient	PCR
176	Yu X, 2015	China	1820	Children	both	PCR
177	Yui I, 2014	Japan	1690	Children	outpatient	EIA, IC
178	Zeng M, 2010	China	351	Children	inpatient	IF
179	Zeng Sz,2015	China	2613	Children	inpatient	PCR
180	Zhang C,2013	China	370	Children	inpatient	PCR
181	Zhang D,2014	China	14237	All ages	both	PCR
182	Zhang G, 2012	China	164	Children	inpatient	PCR
183	Zhang HY, 2009	China	412	Children	inpatient	IF
184	Zhang Q, 2011	China	821	Children	inpatient	Serology,IF
185	Zhang Q,2013	China	707	Children	inpatient	IF
186	Zhang RF,2010	China	894	Children	inpatient	PCR
187	Zhang T, 2014	China	17569	Children	inpatient	IF
188	Zhang XB,2014	China	1726	Children	inpatient	IF
189	Zhang XL,2013	China	42664	Children	inpatient	IF
190	Zhang ZY, 2010	China	1387	Children	inpatient	PCR
191	Zhao B, 2013	China	554	Children	inpatient	PCR
192	Zheng Y,2017	China	80	Children	inpatient	PCR
193	Zhou W,2013	China	273	Children	inpatient	PCR
194	Zou L,2016	China	3843	All ages	both	PCR
195	Zhu R, 2014	China	270	Children	both	IF, Luminex
196	Zhao M,2017	China	128	Adult	inpatient	PCR

References

1. Anders KL, Nguyen HL, Nguyen NM, Thuy NTV, Van NTH, Hieu NT, et al. Epidemiology and Virology of Acute Respiratory Infections During the First Year of Life A Birth Cohort Study in Vietnam. *Ped Infect Dis J.* 2015;34(4):361-70.
2. Arden KE, McErlean P, Nissen MD, Sloots TP, Mackay IM. Frequent detection of human rhinoviruses, paramyxoviruses, coronaviruses, and bocavirus during acute respiratory tract infections. *J Med Virol*. 2006;78(9):1232-40.
3. Arnott A, Vong S, Mardy S, Chu S, Naughtin M, Sovann L, et al. A Study of the Genetic Variability of Human Respiratory Syncytial Virus (HRSV) in Cambodia Reveals the Existence of a New HRSV Group B Genotype. *J Clin Microbiol*. 2011;49(10):3504-13.
4. Baek YH, Choi EH, Song MS, Pascua PN, Kwon HI, Park SJ, et al. Prevalence and genetic characterization of respiratory syncytial virus (RSV) in hospitalized children in Korea. *Arch Virol*. 2012;157(6):1039-50
5. Benet T, Sanchez Picot V, Messaoudi M, Chou M, Eap T, Wang J, et al. Microorganisms Associated with Pneumonia in Children <5 Years of Age in Developing and Emerging Countries: The GABRIEL Pneumonia Multicenter, Prospective, Case-Control Study. *Clin Infect Dis*. 2017;65(4):604-12.
6. Bolisetty S, Wheaton G, Chang AB. Respiratory syncytial virus infection and immunoprophylaxis for selected high-risk children in Central Australia. *Aus J of Rural Health*. 2005;13(5):265-70.
7. Buecher C, Mardy S, Wang W, Duong V, Vong S, Naughtin M, et al. Use of a multiplex PCR/RT-PCR approach to assess the viral causes of influenza-like illnesses in Cambodia during three consecutive dry seasons. *J Med Virol*. 2010;82(10):1762-72.
8. Cai XY, Wang Q, Lin GY, Cai ZW, Lin CX, Chen PZ, et al. Respiratory virus infections among children in South China. *J Med Virol*. 2014;86(7):1249-55.
9. Chan DCW, Chiu WK, Ip PLS. Respiratory syncytial virus and influenza infections among children <=3 years of age with acute respiratory infections in a regional hospital in Hong Kong. *Hong Kong J Paed*. 2007;12(1):15-21+61-2.
10. Chan PKS, Tam WWS, Lee TC, Hon KL, Lee N, Chan MCW, et al. Hospitalization incidence, mortality, and seasonality of common respiratory viruses over a period of 15 years in a developed subtropical city. *Medicine(United States).* 2015;94(46):e2024.
11. Chan PW, Chew FT, Tan TN, Chua KB, Hooi PS. Seasonal variation in respiratory syncytial virus chest infection in the tropics. *Pediatr Pulmonol*. 2002;34(1):47-51.
12. Charles PG, Whitby M, Fuller AJ, Stirling R, Wright AA, Korman TM, et al. The etiology of community-acquired pneumonia in Australia: why penicillin plus doxycycline or a macrolide is the most appropriate therapy. *Clin Infect Dis*. 2008; (10):1513-21.
13. Chaw L, Kamigaki T, Burmaa A, Urtnasan C, Od I, Nyamaa G, et al. Burden of influenza and respiratory syncytial virus infection in pregnant women and infants under 6 months in Mongolia: A prospective cohort study. *PLoS ONE*. 2016;11(2).
14. Chen K, Jia R, Li L, Yang C, Shi Y. The aetiology of community associated pneumonia in children in Nanjing, China and aetiological patterns associated with age and season. *BMC Public Health*. 2015;15(1)
15. Chen X, Zhang ZY, Zhao Y, Liu EM, Zhao XD. Acute lower respiratory tract infections by human metapneumovirus in children in Southwest China: A 2-year study. *Pediatr Pulmonol*. 2010;45(8):824-31.

16. Chen Y, Liu F, Wang C, Zhao M, Deng L, Zhong J, et al. Molecular identification and epidemiological features of human adenoviruses associated with acute respiratory infections in hospitalized children in Southern China, 2012-2013. *PLoS ONE*. 2016;11(5)
17. Chen ZR, Ji W, Wang YQ, Yan YD, Shao XJ, Zhang XL, et al. Etiology of acute bronchiolitis and the relationship with meteorological conditions in hospitalized infants in China. *J Formos Med Assoc*. 2014;113(7):463-9.
18. Chen ZR, Zhu Y, Wang YQ, Zhou WF, Yan YD, Zhu CH, et al. Association of meteorological factors with childhood viral acute respiratory infections in subtropical China: an analysis over 11 years. *Arch Virol*. 2014;159(4):631-9.
19. Chidlow GR, Laing IA, Harnett GB, Greenhill AR, Phuanukoonnon S, Siba PM, et al. Respiratory viral pathogens associated with lower respiratory tract disease among young children in the highlands of Papua New Guinea. *J Clin Virol*. 2012;54(3):235-9.
20. Chiu SS, Chan KH, Chen H, Young BW, Lim W, Wong WH, et al. Virologically confirmed population-based burden of hospitalization caused by respiratory syncytial virus, adenovirus, and parainfluenza viruses in children in Hong Kong. *Pediatr Infect Dis J*. 2010;29(12):1088-92.
21. Cho HJ, Shim SY, Son DW, Sun YH, Tchah H, Jeon IS. Respiratory viruses in neonates hospitalized with acute lower respiratory tract infections. *Pediatr Int*. 2013;55(1):49-53.
22. Choi EH, Lee HJ, Kim SJ, Eun BW, Kim NH, Lee JA, et al. The association of newly identified respiratory viruses with lower respiratory tract infections in Korean children, 2000-2005. *Clin Infect Dis*. 2006;43(5):585-92.
23. Chow A, Ma S, Ling AE, Chew SK. Influenza-associated deaths in tropical Singapore. *Emerg Infect Dis* 2006;12(1):114-21.
24. Chun JK, Lee JH, Kim HS, Cheong HM, Kim KS, Kang C, et al. Establishing a surveillance network for severe lower respiratory tract infections in Korean infants and young children. *Eur J Clin Microbiol Infect Dis*. 2009;28(7):841-4.
25. Chung JY, Han TH, Kim BE, Kim CK, Kim SW, Hwang ES. Human metapneumovirus infection in hospitalized children with acute respiratory disease in Korea. *J Korean Med Sci.* 2006;21(5):838-42
26. Chung JY, Han TH, Kim SW, Hwang ES. Respiratory picornavirus infections in Korean children with lower respiratory tract infections. *Scand J Infect Dis*. 2007;39(3):250-4.
27. Cui B, Zhang D, Pan H, Zhang F, Farrar J, Law F, et al. Viral aetiology of acute respiratory infections among children and associated meteorological factors in southern China. *BMC Infect Dis*. 2015;15(1).
28. Cui G, Zhu R, Qian Y, Deng J, Zhao L, Sun Y, et al. Genetic Variation in Attachment Glycoprotein Genes of Human Respiratory Syncytial Virus Subgroups A and B in Children in Recent Five Consecutive Years. *PLoS ONE*. 2013;8(9).
29. Dang JL, Zhao JJ. Viral respiratory tract infections and their correlation with clinical presentations and outcomes among young children attending emergency department of tertiary care hospital in china. *Biomed Res* (India). 2017;28(5):2327-33.
30. Dapat IC, Shobugawa Y, Sano Y, Saito R, Sasaki A, Suzuki Y, et al. New genotypes within respiratory syncytial virus group B genotype BA in Niigata, Japan. *J Clin Microbiol*. 2010;48(9):3423-7.
31. Dede A, Isaacs D, Torzillo PJ, Wakerman J, Roseby R, Fahy R, et al. Respiratory syncytial virus infections in Central Australia. *J Paediatr Child Health*. 2010;46(1-2):35-9.
32. Do AHL, van Doorn HR, Nghiem MN, Bryant JE, Hoang THt, Do QH, et al. Viral etiologies of acute respiratory infections among hospitalized vietnamese children in Ho Chi Minh City, 2004-2008. *PLoS ONE*. 2011;6(3).

33. Do LA, Bryant JE, Tran AT, Nguyen BH, Tran TT, Tran QH, et al. Respiratory Syncytial Virus and Other Viral Infections among Children under Two Years Old in Southern Vietnam 2009-2010: Clinical Characteristics and Disease Severity. *PLoS ONE*. 2016;11(8):e0160606.
34. Dong W, Chen Q, Hu Y, He D, Liu J, Yan H, et al. Epidemiological and clinical characteristics of respiratory viral infections in children in Shanghai, China. *Arch Virol*. 2016;161(7):1907-13.
35. Etemadi MR, Sekawi Z, Othman N, Lye MS, Moghaddam FY. Circulation of human respiratory syncytial virus strains among hospitalized children with acute lower respiratory infection in Malaysia. *Evol Bioinform*. 2013;2013(9):151-61
36. Fagan P, McLeod C, Baird RW. Seasonal variability of respiratory syncytial virus infection in the Top End of the Northern Territory (2012–2014). *J Paediatr Child Health*. 2017;53(1):43-6.
37. Fan R, Fan C, Zhang J, Wen B, Lei Y, Liu C, et al. Respiratory syncytial virus subtype ON1/NA1/BA9 predominates in hospitalized children with lower respiratory tract infections. *J Med Virol*. 2017;89(2):213-21.
38. Feng L, Li Z, Zhao S, Nair H, Lai S, Xu W, et al. Viral etiologies of hospitalized acute lower respiratory infection patients in China, 2009-2013. *PLoS ONE*. 2014;9 (6) (no pagination)(e99419).
39. Fuller DG, Davie G, Lamb D, Carlin JB, Curtis N. Analysis of respiratory viral coinfection and cytomegalovirus co-isolation in pediatric inpatients*. Pediatr Infect Dis J*. 2005;24(3):195-200
40. Grimwood K, Cohet C, Rich FJ, Cheng S, Wood C, Redshaw N, et al. Risk factors for respiratory syncytial virus bronchiolitis hospital admission in New Zealand. *Epidemiol Infect*. 2008;136(10):1333-41.
41. Guerrier G, Goyet S, Chheng ET, Rammaert B, Borand L, Te V, et al. Acute viral lower respiratory tract infections in cambodian children: Clinical and epidemiologic characteristics. *Pediatr Infect Dis J*. 2013;32(1):e8-e13.
42. Han TH, Chung JY, Kim SW, Hwang ES. Human Coronavirus-NL63 infections in Korean children, 2004-2006. *J Clin Virol*. 2007;38(1):27-31.
43. Hara M, Takao S. Coronavirus infections in pediatric outpatients with febrile respiratory tract infections in Hiroshima, Japan, over a 3-year period. *Jpn J Infect Dis*. 2015;68(6):523-5.
44. Hara M, Takao S, Fukuda S, Shimazu Y, Miyazaki K. Human metapneumovirus infection in febrile children with lower respiratory diseases in primary care settings in Hiroshima, Japan. *Jpn J Infect Dis*. 2008;61(6):500-2.
45. Hara M, Takao S, Shimazu Y, Nishimura T. Three-year study of viral etiology and features of febrile respiratory tract infections in Japanese pediatric outpatients. *Pediatr Infect Dis J*. 2014;33(7):687-92.
46. Harada Y, Kinoshita F, Yoshida LM, Le Nhat M, Suzuki M, Morimoto K, et al. Does respiratory virus coinfection increases the clinical severity of acute respiratory infection among children infected with respiratory syncytial virus? *Pediatr Infect Dis J*.. 2013;32(5):441-5.
47. He Y, Lin GY, Wang Q, Cai XY, Zhang YH, Lin CX, et al. A 3-year prospective study of the epidemiology of acute respiratory viral infections in hospitalized children in Shenzhen, China. *Influenza Other Respi Viruses*. 2014;8(4):443-51.
48. Homaira N, Oei JL, Mallitt KA, Abdel-Latif ME, Hilder L, Bajuk B, et al. High burden of RSV hospitalization in very young children: A data linkage study. *Epidemiol Infect*. 2016;144(8):1612-21.

49. Hu P, Zheng T, Chen J, Zhou T, Chen Y, Xu X, et al. Alternate circulation and genetic variation of human respiratory syncytial virus genotypes in Chengdu, West China, 2009-2014. *J Med Virol*. 2017;89(1):32-40
50. Huang G, Yu D, Mao N, Zhu Z, Zhang H, Jiang Z, et al. Viral Etiology of Acute Respiratory Infection in Gansu Province, China, 2011. *PLoS ONE*. 2013;8 (5) (no pagination)(e64254).
51. Hui DS, Woo J, Hui E, Foo A, Ip M, To KW, et al. Influenza-like illness in residential care homes: a study of the incidence, aetiological agents, natural history and health resource utilisation. *Thorax*. 2008;63(8):690-7.
52. Huo X, Fang B, Liu L, Yu H, Chen H, Zheng J, et al. Clinical and epidemiologic characteristics of respiratory syncytial virus infection among children aged <5 years, Jingzhou city, China, 2011. *J Infect Dis*. 2013;208(SUPPL. 3):S184-S8.
53. Huo X, Qin Y, Qi X, Zu R, Tang F, Li L, et al. Surveillance of 16 respiratory viruses in patients with influenza-like illness in Nanjing, China.*J Med Virol*. 2012;84(12):1980-4
54. Ikematsu H, Takeuchi Y, Rosenlund M, Kawai N, Shimamura R, Hirata M, et al. The post-infection outcomes of influenza and acute respiratory infection in patients above 50years of age in Japan: An observational study. *Influenza Other Respi Viruses*. 2012;6(3):211-7.
55. Jacoby P, Glass K, Moore HC. Characterizing the risk of respiratory syncytial virus in infants with older siblings: A population-based birth cohort study.*Epidemiol Infect*. 2017;145(2):266-71.
56. Ji W, Zhang T, Zhang X, Jiang L, Ding Y, Hao C, et al. The epidemiology of hospitalized influenza in children, a two year population-based study in the People's Republic of China. *BMC Health Serv Res*. 2010;10.
57. Jin Y, Yuan XH, Xie ZP, Gao HC, Song JR, Zhang RF, et al. Prevalence and Clinical Characterization of a Newly Identified Human Rhinovirus C Species in Children with Acute Respiratory Tract Infections.*J Clin Microbiol*. 2009;47(9):2895-900.
58. Jin Y, Zhang RF, Xie ZP, Yan KL, Gao HC, Song JR, et al. Newly identified respiratory viruses associated with acute lower respiratory tract infections in children in Lanzou, China, from 2006 to 2009. *Clin Microbiol Infect*. 2012;18(1):74-80.
59. Ju X, Fang Q, Zhang J, Xu A, Liang L, Ke C. Viral etiology of influenza-like illnesses in Huizhou, China, from 2011 to 2013. *Arch Virol*. 2014;159(8):2003-10
60. Kadji FMN, Okamoto M, Furuse Y, Tamaki R, Suzuki A, Lirio I, et al. Differences in viral load among human respiratory syncytial virus genotypes in hospitalized children with severe acute respiratory infections in the Philippines. *Virol J*. 2016;13(1).
61. Kaida A, Kubo H, Takakura KI, Sekiguchi JI, Yamamoto SP, Kohdera U, et al. Associations between CO-detected respiratory viruses in children with acute respiratory infections. *Jpn J Infect Dis*. 2014;67(6):469-75.
62. Kamigaki T, Aldey PP, Mercado ES, Tan AG, Javier JB, Lupisan SP, et al. Estimates of influenza and respiratory syncytial virus incidences with fraction modeling approach in Baguio City, the Philippines, 2012-2014. *Influenza Other Respi Viruses*. 2017;11(4):311-8
63. Kamigaki T, Chaw L, Tan AG, Tamaki R, Alday PP, Javier JB, et al. Seasonality of influenza and respiratory syncytial viruses and the effect of climate factors in subtropical-tropical asia using influenza-like illness surveillance data, 2010 - 2012. *PLoS ONE*. 2016;11(12).
64. Kaneko M, Watanabe J, Kuwahara M, Ueno E, Hida M, Kinoshita A, et al. Impact of respiratory synctial virus infection as a cause of lower respiratory tract infection in children younger than 3 years of age in Japan. *J Infect*. 2002;44(4):240-3.

65. Khor CS, Sam IC, Hooi PS, Quek KF, Chan YF. Epidemiology and seasonality of respiratory viral infections in hospitalized children in Kuala Lumpur, Malaysia: A retrospective study of 27 years. *BMC Pediatr*. 2012;12.
66. Kim CK, Choi J, Callaway Z, Kim HB, Chung JY, Koh YY, et al. Clinical and epidemiological comparison of human metapneumovirus and respiratory syncytial virus in seoul, Korea, 2003-2008. *J Korean Med Sci*. 2010;25(3):342-7.
67. Kim KY, Han SY, Kim HS, Cheong HM, Kim SS, Kim DS. Human coronavirus in the 2014 winter season as a cause of lower respiratory tract infection. *Yonsei Med J*. 2017;58(1):174-9.
68. Kim MR, Lee HR, Lee GM. Epidemiology of acute viral respiratory tract infections in Korean children.*J Infect*. 2000;41(2):152-8.
69. Kim YJ, Kim DW, Lee WJ, Yun MR, Lee HY, Lee HS, et al. Rapid replacement of human respiratory syncytial virus A with the ON1 genotype having 72 nucleotide duplication in G gene. *Infect Genet Evol*. 2014;26:103-12.
70. Kim YK, Nyambat B, Hong YS, Lee CG, Lee JW, Kilgore PE. Burden of viral respiratory disease hospitalizations among children in a community of Seoul, Republic of Korea, 1995-2005. *Scand J Infect Dis*. 2008;40(11-12):946-53.
71. Ko DH, Hyun J, Kim HS, Kim JS, Song W, Kim HS. Analysis of respiratory viral infections detected using multiplex real-time PCR in Hwaseong, Korea from 2013 to 2015. *Clin Lab*. 2017;63(5-6):1003-7.
72. Koh J, Wong JJ, Sultana R, Wong PPC, Mok YH, Lee JH. Risk factors for mortality in children with pneumonia admitted to the pediatric intensive care unit. *Pediatr Pulmonol*. 2017;52(8):1076-84.
73. Ko FW, Ip M, Chan PK, Fok JP, Chan MC, Ngai JC, et al. A 1-year prospective study of the infectious etiology in patients hospitalized with acute exacerbations of COPD. Chest. 2007;131(1):44-52.
74. Kono J, Jonduo MH, Omena M, Siba PM, Horwood PF. Viruses associated with influenza-like-illnesses in Papua New Guinea, 2010. *J Med Virol*. 2014;86(5):899-904.
75. Kushibuchi I, Kobayashi M, Kusaka T, Tsukagoshi H, Ryo A, Yoshida A, et al. Molecular evolution of attachment glycoprotein (G) gene in human respiratory syncytial virus detected in Japan 2008-2011.*Infect Genet Evol*. 2013;18:168-73
76. Kusuda S, Takahashi N, Saitoh T, Terai M, Kaneda H, Kato Y, et al. Survey of pediatric ward hospitalization due to respiratory syncytial virus infection after the introduction of palivizumab to high-risk infants in Japan. *Pediatr Int*. 2011;53(3):368-73.
77. Lambert SB, Allen KM, Druce JD, Birch CJ, Mackay IM, Carlin JB, et al. Community epidemiology of human metapneumovirus, human coronavirus NL63, and other respiratory viruses in healthy preschool-aged children using parent-collected specimens. *Pediatrics*. 2007;120(4):e929-e37.
78. Lazzaro T, Hogg G, Barnett P. Respiratory syncytial virus infection and recurrent wheeze/asthma in children under five years: An epidemiological survey. *J Paediatr Child Health*. 2007;43(1-2):29-33.
79. Lee SL, Chiu SS, Malik PJ, Chan KH, Wong HS, Lau YL. Is respiratory viral infection really an important trigger of asthma exacerbations in children? *Eur J Pediatr*. 2011;170(10):1317-24.
80. Lee WK, Young BWY. Infectious diseases in children admitted from a residential child care centre. *Hong Kong Med J*. 2006;12(2):119-24.

81. Li HX, Wei QD, Tan AJ, Wang LY. Epidemiological analysis of respiratory viral etiology for influenza-like illness during 2010 in Zhuhai, China. *Virol J*. 2013;10
82. Liao H, Yang Z, Yang C, Tang Y, Liu S, Guan W, et al. Impact of viral infection on acute exacerbation of asthma in out-patient clinics: A prospective study. *J Thorac Dis*. 2016;8(3):505-12.
83. Liao X, Hu Z, Liu W, Lu Y, Chen D, Chen M, et al. New epidemiological and clinical signatures of 18 pathogens from respiratory tract infections based on a 5-year study. *PLoS ONE*. 2015;10(9).
84. Lim FJ, Blyth CC, Fathima P, de Klerk N, Moore HC. Record linkage study of the pathogen-specific burden of respiratory viruses in children. *Influenza Other Respi Viruses*. 2017;11(6):502-10.
85. Lim FJ, Wake ZV, Levy A, Tempone S, Moore HC, Richmond PC, et al. Viral Etiology and the Impact of Codetection in Young Children Presenting With Influenza-Like Illness. *J Pediatr Infect Dis Societ*. 2017;6(3):260-6.
86. Liu CY, Xiao Y, Zhang J, Ren LL, Li JG, Xie ZD, et al. Adenovirus infection in children with acute lower respiratory tract infections in Beijing, China, 2007 to 2012. *BMC Infect Dis.* 2015;15
87. Liu J, Ai H, Xiong Y, Li F, Wen Z, Liu W, et al. Prevalence and correlation of infectious agents in hospitalized children with acute respiratory tract infections in central China. *PLoS ONE*. 2015;10(3).
88. Liu J, Mu Y, Dong W, Yao F, Wang L, Yan H, et al. Genetic variation of human respiratory syncytial virus among children with fever and respiratory symptoms in Shanghai, China, from 2009 to 2012. *Infect Genet Evol*. 2014;27:131-6.
89. Liu T, Li Z, Zhang S, Song S, Julong W, Lin Y, et al. Viral Etiology of acute respiratory tract infections in hospitalized children and adults in Shandong Province, China Other viruses (e.g. pox, papilloma, parvo, reoviridae). *Virol J*. 2015;12(1)
90. Liu W, Chen D, Tan W, Xu D, Qiu S, Zeng Z, et al. Epidemiology and clinical presentations of respiratory syncytial virus subgroups A and B detected with multiplex real-time PCR. *PLoS ONE*. 2016;11(10).
91. Liu WK, Liu Q, De Chen H, Liang HX, Chen XK, Chen MX, et al. Epidemiology of acute respiratory infections in children in Guangzhou: A three-year study. PLoS ONE. 2014;9(5).
92. Liu Y, Liu J, Chen F, Shamsi BH, Wang Q, Jiao F, et al. Impact of meteorological factors on lower respiratory tract infections in children. *J Int Med Res*. 2016;44(1):30-4
93. Lu GL, Li JG, Xie ZD, Liu CY, Guo L, Vernet G, et al. Human metapneumovirus associated with community-acquired pneumonia in children in Beijing, China. *J Med Virol*. 2013;85(1):138-43.
94. Lu L, Yan Y, Yang B, Xiao Z, Feng X, Wang Y, et al. Epidemiological and clinical profiles of respiratory syncytial virus infection in hospitalized neonates in Suzhou, China. *BMC Infect Dis*. 2015;15(1).
95. Lu RJ, Yu XY, Wang W, Duan XJ, Zhang LL, Zhou WM, et al. Characterization of Human Coronavirus Etiology in Chinese Adults with Acute Upper Respiratory Tract Infection by Real-Time RT-PCR Assays. *Plos ONE*. 2012;7(6).
96. Lu YQ, Tong JB, Pei FY, Yang YP, Xu D, Ji MY, et al. Viral Aetiology in Adults with Acute Upper Respiratory Tract Infection in Jinan, Northern China. *Clin Develop Immunol*. 2013.

97. Lu YQ, Wang SF, Zhang LH, Xu C, Bian CR, Wang ZX, et al. Epidemiology of Human Respiratory Viruses in Children with Acute Respiratory Tract Infections in Jinan, China. *Clin Develop Immunol*. 2013.
98. Ma HM, Lee KP, Woo J. Predictors of viral pneumonia: The need for viral testing in all patients hospitalized for nursing home-acquired pneumonia. *Geriatr Gerontol Int*. 2013;13(4):949-57.
99. Malasao R, Okamoto M, Chaimongkol N, Imamura T, Tohma K, Dapat I, et al. Molecular characterization of human respiratory syncytial virus in the Philippines, 2012-2013. *PLoS ONE*. 2015;10 (11) (no pagination)(e0142192).
100. Mermond S, Zurawski V, D'Ortenzio E, Driscoll AJ, Deluca AN, Deloria-Knoll M, et al. Lower respiratory infections among hospitalized children in New Caledonia: A pilot study for the pneumonia etiology research for child health project. Clin Infect Dis. 2012;54(SUPPL. 2):S180-S9.
101. Miyaji Y, Kobayashi M, Sugai K, Tsukagoshi H, Niwa S, Fujitsuka-Nozawa A, et al. Severity of respiratory signs and symptoms and virus profiles in Japanese children with acute respiratory illness. *Microbiol Immunol*. 2013;57(12):811-21.
102. Mizuta K, Abiko C, Aoki Y, Ikeda T, Matsuzaki Y, Itagaki T, et al. Seasonal patterns of respiratory syncytial virus, influenza A virus, human metapneumovirus, and parainfluenza virus type 3 infections on the basis of virus isolation data between 2004 and 2011 in Yamagata, Japan. *Jpn J Dis*. 2013;66(2):140-5.
103. Moore HC, De Klerk N, Keil AD, Smith DW, Blyth CC, Richmond P, et al. Use of data linkage to investigate the aetiology of acute lower respiratory infection hospitalisations in children. J Paediatr Child Health. 2012;48(6):520-8.
104. Moriyama Y, Hamada H, Okada M, Tsuchiya N, Maru H, Shirato Y, et al. Distinctive clinical features of human bocavirus in children younger than 2 years. *Eur J Pediatr*. 2010;169(9):1087-92.
105. Moriyama Y, Hamada H, Okada M, Tsuchiya N, Maru H, Shirato Y, et al. Distinctive clinical features of human bocavirus in children younger than 2 years. *Eur J Pediatr*. 2010;169(9):1087-92.
106. Nathan AM, Rani F, Lee RJY, Zaki R, Westerhout C, Sam IC, et al. Clinical risk factors for life-threatening lower respiratory tract infections in children: A retrospective study in an urban city in Malaysia. *PLoS ONE*. 2014;9(10).
107. Ng KF, Tan KK, Sam ZH, Ting GSS, Gan WY. Epidemiology, clinical characteristics, laboratory findings and severity of respiratory syncytial virus acute lower respiratory infection in Malaysian children, 2008-2013. *J Paediatr Child Health*. 2017;53(4):399-407.
108. Nguyen DNT, Mai LQ, Bryant JE, Hang NLK, Hoa LNM, Nadjm B, et al. Epidemiology and etiology of influenza-like-illness in households in Vietnam; it's not all about the kids! *J Clin Virol*. 2016;82:126-32.
109. Nguyen HKL, Nguyen SV, Nguyen AP, Hoang PMV, Le TT, Nguyen TC, et al. Surveillance of severe acute respiratory infection (SARI) for hospitalized patients in Northern Vietnam, 2011–2014. *Jpn J Infect Dis*. 2017;70(5):522-7.
110. Nguyen VH, Dubot-Peres A, Russell FM, Dance DAB, Vilivong K, Phommachan S, et al. Acute respiratory infections in hospitalized children in Vientiane, Lao PDR - the importance of Respiratory Syncytial Virus. *Sci Rep*. 2017;7.
111. Nishimura N, Nishio H, Lee MJ, Uemura K. The clinical features of respiratory syncytial virus: Lower respiratory tract infection after upper respiratory tract infection due to influenza virus. *Pediatr Int*. 2005;47(4):412-6.

112. Noh JY, Song JY, Cheong HJ, Choi WS, Lee J, Lee JS, et al. Laboratory Surveillance of Influenza-Like Illness in Seven Teaching Hospitals, South Korea: 2011-2012 Season. *PLoS ONE*. 2013;8(5).
113. Nolan T, Borja-Tabora C, Lopez P, Weckx L, Ulloa-Gutierrez R, Lazcano-Ponce E, et al. Prevalence and Incidence of Respiratory Syncytial Virus and Other Respiratory Viral Infections in Children Aged 6 Months to 10 Years With Influenza-like Illness Enrolled in a Randomized Trial. *Clin Infect Dis*. 2015;60(11):E80-E9.
114. O'Grady KAF, Grimwood K, Sloots TP, Whiley DM, Acworth JP, Phillips N, et al. Upper airway viruses and bacteria and clinical outcomes in children with cough. *Pediatr Pulmonol*. 2017;52(3):373-81.
115. Ohno A, Suzuki A, Lupisan S, Galang H, Sombrero L, Aniceto R, et al. Genetic characterization of human respiratory syncytial virus detected in hospitalized children in the Philippines from 2008 to 2012. *J Clin Virol*. 2013;57(1):59-65.
116. Park E, Park PH, Huh JW, Yun HJ, Lee HK, Yoon MH, et al. Molecular and clinical characterization of human respiratory syncytial virus in South Korea between 2009 and 2014. Epidemiol Infect. 2017;145(15):3226-42.
117. Park HW, Lee BS, Kim AR, Yoon HS, Kim BI, Song ES, et al. Epidemiology of respiratory syncytial virus infection in infants born at less than thirty-five weeks of gestational age. Pediatr Infect Dis J. 2012;31(8):e99-e104.
118. Park JY, Park S, Lee SH, Lee MG, Park YB, Oh KC, et al. Microorganisms causing community-acquired acute bronchitis: The role of bacterial infection. *PLoS ONE*. 2016;11(10).
119. Park K, Kim D, Seong J, Shin I, Hong J, Park S, et al. Epidemiological features and genetic variation of human respiratory syncytial virus (HRSV) infection in Chungnam, Korea. *Biomed Res (India).* 2017;28(2):967-72.
120. Paynter S, Ware RS, Lucero MG, Tallo V, Nohynek H, Simões EAF, et al. Poor Growth and Pneumonia Seasonality in Infants in the Philippines: Cohort and Time Series Studies. *PLoS ONE*. 2013;8(6).
121. Qin X, Zhang C, Zhao Y, Zhao X. Genetic variability of subgroup A and B respiratory syncytial virus strains circulating in southwestern China from 2009 to 2011. *Arch Virol*. 2013;158(7):1487-95
122. Qu XW, Duan ZJ, Qi ZY, Xie ZP, Gao HC, Liu WP, et al. Human bocavirus infection, People's Republic of China. *Emerg Infect Dis*. 2007;13(1):165-8
123. Rahman MM, Wong KK, Hanafiah A, Isahak I. Influenza and respiratory syncytial viral infections in Malaysia: Demographic and clinical perspective. Pak J Med Sci. 2014;30(1):161-5.
124. Ranmuthugala G, Brown L, Lidbury BA. Respiratory syncytial virus--the unrecognised cause of health and economic burden among young children in Australia. *Commun Dis Intell Q Rep*. 2011;35(2):177-84.
125. Reeve CA, Whitehall JS, Buettner PG, Norton R, Reeve DM, Francis F. Predicting respiratory syncytial virus hospitalisation in Australian children. J Paediatr Child Health. 2006;42(5):248-52.
126. Ren L, Gonzalez R, Wang Z, Xiang Z, Wang Y, Zhou H, et al. Prevalence of human respiratory viruses in adults with acute respiratory tract infections in Beijing, 2005-2007. Clin Microbiol Infect. 2009;15(12):1146-53.
127. Ren L, Xiao Q, Zhou L, Xia Q, Liu E. Molecular characterization of human respiratory syncytial virus subtype B: A novel genotype of subtype B circulating in China. *J Med Virol*. 2015;87(1):1-9.

128. Saraya T, Kimura H, Kurai D, Ishii H, Takizawa H. The molecular epidemiology of respiratory viruses associated with asthma attacks. *Medicine (United States).* 2017;96(42).
129. Sato M, Saito R, Sakai T, Sano Y, Nishikawa M, Sasaki A, et al. Molecular epidemiology of respiratory syncytial virus infections among children with acute respiratory symptoms in a community over three seasons. *J Clin Microbiol*. 2005;43(1):36-40.
130. Sentilhes AC, Choumlivong K, Celhay O, Sisouk T, Phonekeo D, Vongphrachanh P, et al. Respiratory virus infections in hospitalized children and adults in Lao PDR. *Influenza Other Respir Viruses*. 2013;7(6):1070-8.
131. Seo KH, Bae DJ, Kim JN, Lee HS, Kim YH, Park JS, et al. Prevalence of respiratory viral infections in Korean adult asthmatics with acute exacerbations: Comparison with those with stable state. *Allergy Asthma Immunol Res*. 2017;9(6):491-8
132. Seo YB, Cheong HJ, Song JY, Noh JY, Kim IS, Song DJ, et al. Epidemiologic differences of four major respiratory viruses between children, adolescents, and adults in Korea. *J Infect Chemother*. 2014;20(11):672-7.
133. Shi W, Cui S, Gong C, Zhang T, Yu X, Li A, et al. Prevalence of human parainfluenza virus in patients with acute respiratory tract infections in Beijing, 2011-2014.*Influenza Other Respi Viruses*. 2015;9(6):305-7.
134. Shobugawa Y, Saito R, Sano Y, Zaraket H, Suzuki Y, Kumaki A, et al. Emerging genotypes of human respiratory syncytial virus subgroup A among patients in Japan. *J Clin Microbiol*. 2009;47(8):2475-82
135. Song J, Zhang Y, Wang H, Shi J, Sun L, Zhang X, et al. Emergence of ON1 genotype of human respiratory syncytial virus subgroup A in China between 2011 and 2015. *Sci Rep*. 2017;7(1).
136. Sugaya N, Mitamura K, Nirasawa M, Takahashi K. The impact of winter epidemics of influenza and respiratory syncytial virus on paediatric admissions to an Urban General Hospital. *J Med Virol*. 2000;60(1):102-6.
137. Sun HQ, Sun QF, Jiang WJ, Chen ZR, Huang L, Wang MJ, et al. Prevalence of rhinovirus in wheezing children: a comparison with respiratory syncytial virus wheezing. *Brazil J Infect Dis*. 2016;20(2):179-83.
138. Sung RYT, Chan PKS, Tsen T, Li AM, Lam WY, Yeung ACM, et al. Identification of viral and atypical bacterial pathogens in children hospitalized with acute respiratory infections in Hong Kong by multiplex PCR assays. *J Med Virol*. 2009;81(1):153-9.
139. Suzuki A, Lupisan S, Furuse Y, Fuji N, Saito M, Tamaki R, et al. Respiratory viruses from hospitalized children with severe pneumonia in the Philippines. BMC Infect Dis. 2012;12
140. Takeyama A, Hashimoto K, Sato M, Sato T, Tomita Y, Maeda R, et al. Clinical and epidemiologic factors related to subsequent wheezing after virus-induced lower respiratory tract infections in hospitalized pediatric patients younger than 3 years. *Eur J Pediatr*. 2014;173(7):959-66.
141. Tan BH, Lim EAS, Seah SGK, Loo LH, Tee NWS, Lin RTP, et al. The incidence of human bocavirus infection among children admitted to hospital in Singapore. J Med *Virol*. 2009;81(1):82-9.
142. Tan W, Zhao Y, Lu R, Zhu N. Comparison of viral and epidemiological profiles among hospitalized children with severe acute respiratory diseases in Beijing and Shanghai. *J Clin Virol*. 2016;82 (Supplement 1):S111-S2.
143. Tang LF, Wang TL, Tang HF, Chen ZM. Viral pathogens of acute lower respiratory tract infection in China. *Indian Pediatr*. 2008;45(12):971-5

144. Tian DD, Jiang R, Chen XJ, Ye Q. Meteorological factors on the incidence of MP and RSV pneumonia in children. *PLoS ONE*. 2017;12(3).
145. Tran DN, Pham TMH, Ha MT, Tran TTL, Dang TKH, Yoshida LM, et al. Molecular Epidemiology and Disease Severity of Human Respiratory Syncytial Virus in Vietnam. *PLoS ONE*. 2013;8(1)
146. Tran T, Chien BT, Papadakis G, Druce J, Birch C, Chibo D, et al. Respiratory virus laboratory pandemic planning and surveillance in central Viet Nam, 2008-2010. *Western Pac Surveill Response J*. 2012;3(3):49-56
147. Trenholme AA, Best EJ, Vogel AM, Stewart JM, Miller CJ, Lennon DR. Respiratory virus detection during hospitalisation for lower respiratory tract infection in children under 2 years in South Auckland, New Zealand. *J Paediatr Child Health*. 2017;53(6):551-5
148. Tsukagoshi H, Yokoi H, Kobayashi M, Kushibuchi I, Okamoto-Nakagawa R, Yoshida A, et al. Genetic analysis of attachment glycoprotein (G) gene in new genotype ON1 of human respiratory syncytial virus detected in Japan. *Microbiol Immunol*. 2013;57(9):655-9
149. Tuan TA, Thanh TT, Hai NTT, Tinh LBB, Kim LTN, Do LAH, et al. Characterization of hospital and community-acquired respiratory syncytial virus in children with severe lower respiratory tract infections in Ho Chi Minh City, Vietnam, 2010. *Influenza Other Respi Viruses.* 2015;9(3):110-9.
150. Vong S, Guillard B, Borand L, Rammaert B, Goyet S, Te V, et al. Acute lower respiratory infections in ≥5 year -old hospitalized patients in Cambodia, a low-income tropical country: Clinical characteristics and pathogenic etiology. *BMC Infect Dis*. 2013;13(1).
151. Wang D, Chen L, Ding Y, Zhang J, Hua J, Geng Q, et al. Viral etiology of medically attended influenza-like illnesses in children less than five years old in Suzhou, China, 2011-2014. *J Med Virol*. 2016;88(8):1334-40
152. Wang HP, Zheng YJ, Deng JK, Wang WJ, Liu P, Yang FH, et al. Prevalence of respiratory viruses among children hospitalized from respiratory infections in Shenzhen, China. *Virol J*. 2016;13.
153. Wang W, Cavailler P, Ren P, Zhang J, Dong W, Yan H, et al. Molecular monitoring of causative viruses in child acute respiratory infection in endemo-epidemic situations in Shanghai. *J Clin Virol*. 2010;49(3):211-8
154. Wang Y, Chen Z, Yan YD, Guo H, Chu C, Liu J, et al. Seasonal distribution and epidemiological characteristics of human metapneumovirus infections in pediatric inpatients in Southeast China. *Arch Virol*. 2013;158(2):417-24.
155. Wang Y, Hao C, Ji W, Yan Y, Shao X, Xu J. Bronchiolitis associated with mycoplasma pneumoniae in infants in Suzhou China between 2010 and 2012. *Sci Rep*. 2015;5
156. Watson M, Gilmour R, Menzies R, Ferson M, McIntyre P, New South Wales Pneumococcal N. The association of respiratory viruses, temperature, and other climatic parameters with the incidence of invasive pneumococcal disease in Sydney, Australia. *Clin Infect Dis*. 2006;42(2):211-5.
157. Wei L, Liu W, Zhang XA, Liu EM, Wo Y, Cowling BJ, et al. Detection of viral and bacterial pathogens in hospitalized children with acute respiratory Illnesses, Chongqing, 2009-2013. *Medicine (United States)*. 2015;94(16).
158. Wertheim HFL, Nadjm B, Thomas S, Agustiningsih, Malik S, Nguyen DNT, et al. Viral and atypical bacterial aetiologies of infection in hospitalised patients admitted with clinical suspicion of influenza in Thailand, Vietnam and Indonesia. *Influenza Other Respi Viruses*. 2015;9(6):315-22.

159. Wu Z, Li Y, Gu J, Zheng H, Tong Y, Wu Q. Detection of viruses and atypical bacteria associated with acute respiratory infection of children in Hubei, China. *Respirology*. 2014;19(2):218-24.
160. Xia Q, Zhou L, Peng C, Hao R, Ni K, Zang N, et al. Detection of respiratory syncytial virus fusion protein variants between 2009 and 2012 in China. *Arch Virol*. 2014;159(5):1089-98.
161. Xiang Z, Gonzalez R, Ren L, Xiao Y, Chen L, Zhang J, et al. Prevalence and clinical characteristics of human respiratory syncytial virus in Chinese adults with acute respiratory tract infection. *J Med Virol*. 2013;85(2):348-53.
162. Xiao Q, Zheng S, Zhou L, Ren L, Xie X, Deng Y, et al. Impact of human rhinovirus types and viral load on the severity of illness in hospitalized children with lower respiratory tract infections. *Pediatr Infect Dis J.* 2015;34(11):1187-92.
163. Xie M, Ma Q, Chen X, Lu X, Zhong B. Epidemiological characteristics of seven respiratory viruses among pediatric patients with acute respiratory tract infections from 2011 to 2015 in Dongguan, Southern China. *Brazil J Infect Dis*. 2017;21(4):486-8.
164. Xu L, He X, Zhang Dm, Feng Fs, Wang Z, Guan Ll, et al. Surveillance and Genome Analysis of Human Bocavirus in Patients with Respiratory Infection in Guangzhou, China. *PLoS ONE*. 2012;7(9).
165. Yamaguchi M, Sano Y, Dapat IC, Saito R, Suzuki Y, Kumaki A, et al. High frequency of repeated infections due to emerging genotypes of human respiratory syncytial viruses among children during eight successive epidemic seasons in Japan. *J Clin Microbiol*. 2011;49(3):1034-40
166. Yan XL, Li YN, Tang YJ, Xie ZP, Gao HC, Yang XM, et al. Clinical characteristics and viral load of respiratory syncytial virus and human metapneumovirus in children hospitaled for acute lower respiratory tract infection. *J Med Virol*. 2017;89(4):589-97.
167. Yan Y, Huang L, Wang M, Wang Y, Ji W, Zhu C, et al. Clinical and epidemiological profiles including meteorological factors of low respiratory tract infection due to human rhinovirus in hospitalized children. *Ital J Pediatr*. 2017;43(1).
168. Yang L, Chan KH, Suen LKP, Chan KP, Wang X, Cao P, et al. Age-specific epidemic waves of influenza and respiratory syncytial virus in a subtropical city. *Sci Rep*. 2015;5.
169. Yasuno T, Shimizu T, Maeda Y, Yamasaki A, Amaya E, Kawakatsu H. Wheezing illness caused by respiratory syncytial virus and other agents. Pediatr Int. 2008;50(4):500-5.
170. Ye C, Zhu W, Yu J, Li Z, Fu Y, Lan Y, et al. Viral pathogens among elderly people with acute respiratory infections in Shanghai, China: Preliminary results from a laboratory-based surveillance, 2012-2015. *J Med Virol*. 2017;89(10):1700-6.
171. Ye Q, Fu JF, Mao JH, Shang SQ. Haze is a risk factor contributing to the rapid spread of respiratory syncytial virus in children. *Environ Sci Pollut R*. 2016;23(20):20178-85.
172. Yoshida A, Kiyota N, Kobayashi M, Nishimura K, Tsutsui R, Tsukagoshi H, et al. Molecular epidemiology of the attachment glycoprotein (G) gene in respiratory syncytial virus in children with acute respiratory infection in Japan in 2009/2010. *J Med Microbiol*. 2012;61(6):820-9.
173. Yoshida LM, Suzuki M, Nguyen HA, Le MN, Vu TD, Yoshino H, et al. Respiratory syncytial virus: Co-infection and paediatric lower respiratory tract infections. *Euro Respi J*. 2013;42(2):461-9.

174. Yoshihara K, Le MN, Okamoto M, Wadagni ACA, Nguyen HA, Toizumi M, et al. Association of RSV-A ON1 genotype with Increased Pediatric Acute Lower Respiratory Tract Infection in Vietnam. *Sci Rep*. 2016;6.
175. Yu X, Kou Y, Xia D, Li J, Yang X, Zhou Y, et al. Human respiratory syncytial virus in children with lower respiratory tract infections or influenza-like illness and its co-infection characteristics with viruses and atypical bacteria in Hangzhou, China. *J Clin Virol*. 2015;69:1-6.
176. Yu X, Lu R, Wang Z, Zhu N, Wang W, Julian D, et al. Etiology and clinical characterization of respiratory virus infections in adult patients attending an emergency department in beijing. *PLoS ONE*. 2012;7(2).
177. Yui I, Fujino M, Sawada A, Nakayama T. Novel clinical features of recurrent human respiratory syncytial virus infections. *J Med Virol*. 2014;86(9):1629-38.
178. Zeng M, Zhu QR, Wang XH, Yu H, Shen J. Human bocavirus in children with respiratory tract infection in Shanghai: A retrospective study. *World J Pediatr*. 2010;6(1):65-70.
179. Zeng SZ, Xiao NG, Zhong LL, Yu T, Zhang B, Duan ZJ. Clinical features of human metapneumovirus genotypes in children with acute lower respiratory tract infection in Changsha, China. *J Med Virol*. 2015;87(11):1839-45.
180. Zhang C, Zhu N, Xie Z, Lu R, He B, Liu C, et al. Viral Etiology and Clinical Profiles of Children with Severe Acute Respiratory Infections in China. *PLoS ONE*. 2013;8(8).
181. Zhang D, He Z, Xu L, Zhu X, Wu J, Wen W, et al. Epidemiology characteristics of respiratory viruses found in children and adults with respiratory tract infections in southern China. *Int J Infect Dis*. 2014;25:159-64.
182. Zhang GC, Hu YW, Wang HP, Zhang L, Bao YX, Zhou XM. High Incidence of Multiple Viral Infections Identified in Upper Respiratory Tract Infected Children under Three Years of Age in Shanghai, China. *PLoS ONE*. 2012;7(9)
183. Zhang HY, Li ZM, Zhang GL, Diao TT, Cao CX, Sun HQ. Respiratory viruses in hospitalized children with acute lower respiratory tract infections in Harbin, China. *Jpn J Infect Dis*. 2009;62(6):458-60.
184. Zhang Q, Guo Z, MacDonald NE. Vaccine preventable community-acquired pneumonia in hospitalized children in Northwest China. *Pediatr Infect Dis J*. 2011;30(1):7-10.
185. Zhang QL, Guo ZQ, Bai ZJ, MacDonald NE. A 4 year prospective study to determine risk factors for severe community acquired pneumonia in children in southern China. *Pediatr Pulmonol*. 2013;48(4):390-7.
186. Zhang RF, Jin Y, Xie ZP, Liu N, Yan KL, Gao HC, et al. Human respiratory syncytial virus in children with acute respiratory tract infections in China. *J Clin Microbiol*. 2010;48(11):4193-9.
187. Zhang T, Zhu Q, Zhang X, Ding Y, Steinhoff M, Black S, et al. Clinical characteristics and direct medical cost of respiratory syncytial virus infection in children hospitalized in Suzhou, China. *Pediatr Infect Dis J*. 2014;33(4):337-41.
188. Zhang XB, Liu LJ, Qian LL, Jiang GL, Wang CK, Jia P, et al. Clinical characteristics and risk factors of severe respiratory syncytial virus-associated acute lower respiratory tract infections in hospitalized infants. *World J Pediatr*. 2014;10(4):360-4.
189. Zhang XL, Shao XJ, Wang J, Guo WL. Temporal characteristics of respiratory syncytial virus infection in children and its correlation with climatic factors at a public pediatric hospital in Suzhou. *J Clin Virol*. 2013;58(4):666-70.

190. Zhang ZY, Du LN, Chen X, Zhao Y, Liu EM, Yang XQ, et al. Genetic variability of respiratory syncytial viruses (RSV) Prevalent in southwestern china from 2006 to 2009: Emergence of subgroup B and A RSV as dominant strains. *J Clin Microbiol*. 2010;48(4):1201-7.
191. Zhao B, Yu X, Wang C, Teng Z, Wang C, Shen J, et al. High Human Bocavirus Viral Load Is Associated with Disease Severity in Children under Five Years of Age. *PLoS ONE*. 2013;8(4).
192. Zhao M, Song B, Liu Y, Pu Z, Yu H. Etiological classification and clinical research on community-acquired pneumonia in Yantai, China. *Biomed Res (India).* 2017;28(8):3501-6.
193. Zheng YX, Liu L, Wang SH, Li ZL, Hou M, Li JL, et al. Prevailing genotype distribution and characteristics of human respiratory syncytial virus in northeastern China. *J Med Virol*. 2017;89(2):222-33.
194. Zhou W, Lin F, Teng L, Li H, Hou J, Tong R, et al. Prevalence of herpes and respiratory viruses in induced sputum among hospitalized children with non typical bacterial community-acquired pneumonia. *PLoS ONE*. 2013;8 (11) (no pagination)(e79477)
195. Zhu RN, Song QW, Qian Y, Zhao LQ, Deng J, Wang F, et al. Virus profile in children with acute respiratory infections with various severities in Beijing, China. *Chinese Med J.* 2014;127(21):3706-11.196
196. Zou LR, Yi L, Wu J, Song YC, Huang GF, Zhang X, et al. Evolution and Transmission of Respiratory Syncytial Group A (RSV-A) Viruses in Guangdong, China 2008-2015. *Front Microbiol*. 2016;7

Supplementary Table 2

Supplementary table 2.RSV genotypes circulated in the WPRO countries

Country	Location/State	Year	Genotype	
			RSV A	RSV B
China				
	Chongqing, Southwestern China	2006	GA2*	GB3, GB1
		2007	GA2*	
		2008		BA*
		2009		BA*, GB3, GB1
		2009-2011	GA2	*GB2, BA
		2009-2010	NA1*	BA
		2010-2011	NA1*, NA3, NA4	BA, GB3
		2011-2012	NA1, NA3, NA4	GB5

		2012-2013	NA1, GA1, ON1	BA9, GB5
		2013-2014	NA1	
	Lanzhou, Northwest China	2006-2007	GA2*	
		2007-2008	GA2*	BA
		2008-2009	GA2	BA*, GB2, GB3
	Beijing, Northern China	2007-2008	NA1, NA4**	SAB4
		2008-2009	NA1*	BA9, CB1**, SAB4
		2009-2010	NA1*	BA9, CB1, Bac**
		2010-2011	NA1, NA3**	BA9, CB1, BAc
		2011-2012	NA1, NA4, ON1	BA9, CB1
		2012-2013		B
		2013-2014	ON1*, NA1	
	Shanghai, China	2009-2010	NA1*,	BA9*, CB1**, Bac
		2010-2011	NA1*, ON1	BA9
		2011-2012	NA1*, NA4, NA3	BA9
		2012	NA1, NA4	BA9*
	Zhejiang, Eastern China	2011-2013	NA1*, NA3, ON1	CB1, BA10, BA11**
	Chengdu, Western China	2010	NA1	BA9, BAC, CB-1
		2011	NA1	BA9, BAC, CB-1
		2012	NA1, ON1	BA9, CB1
		2013	ON1	BA9
		2014		BA9
	Changzhou, Eastern China	2014	ON1, NA1	BA9*,, GB2
Japan				

	Niigata, Japan	2001-2002	GA5*, GA7	SAB3, SAB2
		2002-2003	GA5, GA7	BA (BA2, BA5)*, GB3, GB4
		2003-2004	GA5*, GA2	BA (BA4, BA5, BA6)
		2004-2005	GA5, NA1*	BA (BA4, BA7), GB3
		2005-2006	GA5, NA1,NA2*	BA4, BA7*, BA8, BA9
		2006-2007	NA1, NA2*	BA8*, BA9
		2007-2008	GA5, NA2	BA10*, BA7, BA8, BA9
		2008-2009	NA1, NA2	BA9*, BA10
	Kanagawa	2005-2006	GA2	BA
	Aomori(northern Japan), Gunma (central Japan), Kumamoto(southern Japan)	2009-2010	GA2*	BA
	Chiba, Tochigi, Yamaguchi, Kanagawa, Japan	2012-2013	ON1	
	Tochigi, Japan	2008-2011	NA1	BA1, GB2
Malaysia	Selangor	2009	NA1	BA9, BA10*, BA4
	Kuala Lumpur	1994	GA2, GA3, GA7, NA1	
		1995	GA2, GA7	SAB3
		1996	GA2	
		1998	GA2, GA7	
		1999	GA2	SAB3, BA
		2000	GA7, NA1	BA
		2002	NA2	BA
		2003	GA2, GA5, NA2	BA

		2004	GA5, NA1, NA2	
		2005	GA5, NA2	SAB4
		2006	GA2, NA1	BA
		2007	GA5, GA7, NA1, NA2	BA
		2008	GA5, NA1	
		2009	NA1	BA
		2010	GA5, NA1	
		2011	NA1, ON1	BA
Philippines	Tacloban city, Leyte Island	2008-2012	NA1	BA9
	Biliran, Leyte, Palawan, Metro Manila	2012	ON1*, NA1	BA9*, GB2
	Biliran	2012-2013	NA1, ON1	BA9*
South Korea		2010-2011	NA1	
		2011-2012	NA1*, ON1, GA5	
		2012-2013	ON1*	
	Cheongju	2008-2009	NA1, GA5	BA10*, BA9, BA7, CBB
		2009-2010	NA1, GA5, CBA	BA10, BA9, BA11, CBB
	Gyeonggi	2009-2014	NA1*, ON1, GA1, GA5	BA9,B A10

	Chungnam	2011-2014	NA1, ON1*, GA5	BA4
Vietnam	Ho Chi Minh	2009-2010	NA1, GA5	BA4, BA9, BA10
		2010-2011	GA2*	BA9, BA10
	Khan Hoa, Nha thrang	2010-2012	NA1, ON1	
Cambodia	Takeo, Kampong Cham, Battambang,Siem Reap, Phnom Penh	2005		BA7*
		2006	NA1*, GA2	
		2007	NA1*, GA5	BA10
		2008	NA1	BA10*, SAB4**
		2009		BA10*, SAB4**

Note: * =dominant genotypes, **=new genotypes

Supplementary table 3. PRISMA Checklist

Section/topic	#	Checklist item	Reported on page #
**TITLE**			
Title	1	Identify the report as a systematic review, meta-analysis, or both.	1
**ABSTRACT**			
Structured summary	2	Provide a structured summary including, as applicable: background; objectives; data sources; study eligibility criteria, participants, and interventions; study appraisal and synthesis methods; results; limitations; conclusions and implications of key findings; systematic review registration number.	2
**INTRODUCTION**			

Rationale	3	Describe the rationale for the review in the context of what is already known.	3
Objectives	4	Provide an explicit statement of questions being addressed with reference to participants, interventions, comparisons, outcomes, and study design (PICOS).	3
**METHODS**			
Protocol and registration	5	Indicate if a review protocol exists, if and where it can be accessed (e.g., Web address), and, if available, provide registration information including registration number.	N/A
Eligibility criteria	6	Specify study characteristics (e.g., PICOS, length of follow-up) and report characteristics (e.g., years considered, language, publication status) used as criteria for eligibility, giving rationale.	4
Information sources	7	Describe all information sources (e.g., databases with dates of coverage, contact with study authors to identify additional studies) in the search and date last searched.	4
Search	8	Present full electronic search strategy for at least one database, including any limits used, such that it could be repeated.	4
Study selection	9	State the process for selecting studies (i.e., screening, eligibility, included in systematic review, and, if applicable, included in the meta-analysis).	4
Data collection process	10	Describe method of data extraction from reports (e.g., piloted forms, independently, in duplicate) and any processes for obtaining and confirming data from investigators.	4-5
Data items	11	List and define all variables for which data were sought (e.g., PICOS, funding sources) and any assumptions and simplifications made.	N/A
Risk of bias in individual studies	12	Describe methods used for assessing risk of bias of individual studies (including specification of whether this was done at the study or outcome level), and how this information is to be used in any data synthesis.	N/A
Summary measures	13	State the principal summary measures (e.g., risk ratio, difference in means).	5
Synthesis of results	14	Describe the methods of handling data and combining results of studies, if done, including measures of consistency (e.g., I^2) for each meta-analysis.	5

Section/topic	#	Checklist item	Reported on page #
Risk of bias across studies	15	Specify any assessment of risk of bias that may affect the cumulative evidence (e.g., publication bias, selective reporting within studies).	N/A
Additional analyses	16	Describe methods of additional analyses (e.g., sensitivity or subgroup analyses, meta-regression), if done, indicating which were pre-specified.	N/A
**RESULTS**			
Study selection	17	Give numbers of studies screened, assessed for eligibility, and included in the review, with reasons for exclusions at each stage, ideally with a flow diagram.	Figure 1
Study characteristics	18	For each study, present characteristics for which data were extracted (e.g., study size, PICOS, follow-up period) and provide the citations.	Suppl. table 1
Risk of bias within studies	19	Present data on risk of bias of each study and, if available, any outcome level assessment (see item 12).	N/A
Results of individual studies	20	For all outcomes considered (benefits or harms), present, for each study: (a) simple summary data for each intervention group (b) effect estimates and confidence intervals, ideally with a forest plot.	N/A
Synthesis of results	21	Present results of each meta-analysis done, including confidence intervals and measures of consistency.	Table 1
Risk of bias across studies	22	Present results of any assessment of risk of bias across studies (see Item 15).	N/A
Additional analysis	23	Give results of additional analyses, if done (e.g., sensitivity or subgroup analyses, meta-regression [see Item 16]).	N/A
**DISCUSSION**			
Summary of evidence	24	Summarize the main findings including the strength of evidence for each main outcome; consider their relevance to key groups (e.g., healthcare providers, users, and policy makers).	8-13
Limitations	25	Discuss limitations at study and outcome level (e.g., risk of bias), and at review-level (e.g., incomplete retrieval of identified research, reporting bias).	12-13
Conclusions	26	Provide a general interpretation of the results in the context of other evidence, and implications for future research.	13
**FUNDING**			
Funding	27	Describe sources of funding for the systematic review and other support (e.g., supply of data); role of funders for the systematic review.	13

From: Moher D, Liberati A, Tetzlaff J, Altman DG, The PRISMA Group (2009). Preferred Reporting Items for Systematic Reviews and Meta-Analyses: The PRISMA Statement. PLoS Med 6(6):

e1000097. doi:10.1371/journal.pmed1000097
For more information, visit: **www.prisma-statement.org**
